# New Insights into Complex PTSD Treatment: Focus on TAAR1 Agonists

**DOI:** 10.3390/biomedicines13122972

**Published:** 2025-12-03

**Authors:** David-Mandl V. Tseilikman, Vadim E. Tseilikman, Vladislav A. Shatilov, Daria A. Obukhova, Ilya S. Zhukov, Ivan V. Yatsyk, Victoria A. Maistrenko, Vladimir A. Shipelin, Nikita V. Trusov, Marina N. Karpenko, Olga B. Tseilikman, Raul R. Gainetdinov, Jurica Novak

**Affiliations:** 1 Faculty of Fundamental Medicine, Chelyabinsk State University, 454001 Chelyabinsk, Russia; 2 Scientific and Innovation Activity Management, South Ural State University, 454080 Chelyabinsk, Russia; 3 Institute of Medicine and Medical Technologies, Novosibirsk State University, 630090 Novosibirsk, Russia; 4 Institute of Experimental Medicine, 197376 Saint Petersburg, Russia; 5 Institute of Translational Biomedicine, St. Petersburg State University, 199034 Saint Petersburg, Russia; 6 System Biology Department, Institute of Cytology and Genetics of the Siberian Branch of the Russian Academy of Sciences, 630090 Novosibirsk, Russia; 7 Federal Research Centre of Nutrition and Biotechnology, 109240 Moscow, Russia; 8 Y. M. Lopukhin Federal State Budgetary Scientific Research Center, 119435 Moscow, Russia; 9 Centre for Informatics and Computing, Ruđer Bošković Institute, Bijenička Cesta 54, 10000 Zagreb, Croatia

**Keywords:** complex PTSD, monoamines, hippocampus, striatum, agonist, TAAR1, anxiety, depression

## Abstract

**Background/Objectives:** The therapeutic potential of selective trace amine-associated receptor 1 (TAAR1) agonists has been established in multiple animal models of depression and anxiety. PTSD is a debilitating psychiatric disorder frequently characterized by anxiety and often comorbid with major depressive disorder. Complex PTSD represents an even more severe clinical presentation, emerging from prolonged or repeated exposure to traumatic events. Recent studies indicate that TAAR1 agonists can attenuate anxiety-like behaviors in experimental models of PTSD; however, the molecular mechanisms underlying this effect remain poorly understood. In this study, we evaluated whether TAAR1 agonism modulates PTSD-related neurochemical and molecular changes within the hippocampus and striatum. **Methods:** Post-traumatic stress was modeled using predator stress, a validated experimental paradigm relevant to complex PTSD. Treatment consisted of intraperitoneal administration of the TAAR1 agonist LK00764. Monoamine neurotransmitters and their metabolites were quantified, and the expression of genes implicated in noradrenergic, dopaminergic, and serotonergic signaling pathways was assessed. In addition, gene network reconstruction was performed using artificial intelligence to identify TAAR1-dependent regulatory interactions. **Results:** Treatment with a TAAR1 agonist fully prevented behavioral abnormalities in the experimental model of complex PTSD. Neurochemical analyses revealed decreased 5-HT levels in the hippocampus and reduced dopamine and metabolite concentrations in the striatum following TAAR1 agonism. Moreover, TAAR1 activation was associated with increased expression of the neurotrophic factor BDNF in the striatum. Gene network reconstruction identified a distinct molecular hub within the PTSD network, comprising TAAR1-coexpressed genes, their encoded proteins, and interconnected signaling pathways, suggesting a tightly regulated feedback loop. **Conclusions:** These findings provide novel evidence that TAAR1 agonists exert protective effects against complex PTSD-related behavioral and neurochemical abnormalities. The reconstructed TAAR1-centered gene network offers mechanistic insight into receptor-dependent regulation of monoaminergic signaling and neuroplasticity, supporting further exploration of TAAR1 agonists as promising therapeutic candidates for PTSD.

## 1. Introduction

Post-traumatic stress disorder (PTSD) is a severe stress-related psychiatric condition characterized by complex pathophysiology and multi-organ involvement, including profound alterations in brain function [1,2,3,4,5]. The dysregulation of monoamine neurotransmitters, together with abnormalities in the activity of enzymes responsible for their metabolism, represents a major neurochemical hallmark of PTSD [6]. In particular, norepinephrine (NE), dopamine (DA), and serotonin (5-hydroxytryptamine, 5-HT) are critically implicated, as these neurotransmitters regulate circuits underlying core PTSD-related symptoms such as anxiety, hypervigilance, and maladaptive fear responses [7,8]. Both clinical studies and animal models consistently reveal aberrant interactions among these monoaminergic systems during the progression of PTSD [9,10,11,12,13,14,15]. Nevertheless, the molecular mechanisms that lead to such dysregulation remain poorly understood.

Beyond the classical monoamines, another class of endogenous biogenic amines, termed trace amines, exerts neuromodulatory actions with significant functional overlap [16]. Historically described as “false neurotransmitters,” trace amines are capable of influencing the release, reuptake, and signaling dynamics of 5-HT, DA, and NE, thereby shaping a range of behavioral outcomes [17]. Their physiological effects are mediated through a distinct family of G protein-coupled receptors, the trace amine-associated receptors (TAARs), of which nine subtypes (TAAR1–TAAR9) have been identified to date [18,19,20,21,22].

Among these, TAAR1 has received particular attention due to its established role in the neurobiology of major psychiatric and behavioral disorders, including schizophrenia, addiction, mood disorders, and stress-related conditions [23,24,25]. Notably, TAAR1 is co-localized with dopamine D2 receptors on neuronal membranes, suggesting an important modulatory role in dopaminergic signaling [26,27,28,29,30]. Furthermore, TAAR1 expression is enriched in several brain regions tightly linked to psychopathology—such as the ventral tegmental area (VTA), striatum, prefrontal cortex, amygdala, basal ganglia, and hippocampus [31]. These anatomical and molecular features have laid the foundation for therapeutic exploration of TAAR1 ligands in neuropsychiatric disease.

In recent years, the therapeutic promise of TAAR1 activation has been demonstrated across multiple psychiatric contexts. Clinical trials have shown that ulotaront (SEP-363856), a novel TAAR1 agonist, produces significant efficacy in schizophrenia and major depressive disorder [32,33]. Preclinical research further supports anxiolytic, antimanic-like, and antidepressant-like effects of TAAR1 agonists, positioning this receptor as a promising therapeutic target [33].

Of particular relevance to PTSD, early preclinical studies have revealed that both full and partial TAAR1 agonists ameliorate pathological behaviors linked to trauma-related stress [34]. In the single prolonged stress (SPS) paradigm, the partial agonist RO5263397 attenuated anxiety-like behavior in the elevated plus maze (EPM) and improved impaired fear extinction [35]. Chronic treatment with the agonist RO5166017 reduced pathological fear learning in the stress-enhanced fear learning (SEFL) model [36]. These pioneering findings highlight TAAR1 agonism as a promising pharmacological strategy in trauma-related disorders.

Building upon this foundation, the present study advances the investigation of TAAR1 agonists in the context of complex post-traumatic stress disorder (CPTSD), a severe condition arising from chronic or repeated trauma exposure [34,37]. The prevalence of CPTSD varies substantially across trauma-exposed populations, with the highest rates observed among survivors of domestic violence and sexual abuse and among combatants, and the lowest rates reported in emergency service personnel [37]. Recognized as a distinct diagnostic category in ICD-11, CPTSD exhibits partially overlapping yet distinguishable features from PTSD and borderline personality disorder (BPD) [37]. To our knowledge, this is the first study to evaluate TAAR1 agonists within the chronic predator stress model of CPTSD. Here, we examined the impact of TAAR1 activation on monoaminergic neurotransmitters and their metabolites, as well as expression of genes implicated in neurotransmitter metabolism, intracellular signaling, and neuroplasticity in the hippocampus and striatum regions where TAAR1 is prominently expressed. Additionally, we attempted to reconstruct gene networks underlying TAAR1-dependent signaling mechanisms, thereby providing novel insights into the molecular framework through which TAAR1 modulation may confer therapeutic benefit in trauma- and stress-related disorders.

## 2. Materials and Methods

### 2.1. Animals

All experiments were conducted on adult male Wistar rats. The animals were group-housed (2–3 per cage) in standard cages under controlled environmental conditions. Food (standard laboratory rodent diet) and water were available ad libitum. Housing conditions were maintained at a constant temperature of 22–25 °C and relative humidity of approximately 55%. A 12:12 h light–dark cycle was applied, with illumination from 07:00 to 19:00. All experimental procedures were carried out in compliance with the Guide for the Care and Use of Laboratory Animals (National Research Council, U.S., 8th edition, 2011), and protocols received prior approval from the Institutional Animal Care and Use Committee of the Institute of Experimental Medicine, Saint Petersburg, Russia.

### 2.2. CPTSD Model

A validated chronic predator stress paradigm was employed to induce a complex PTSD-like phenotype in rats, as previously described [38]. Fresh sand containing cat urine was collected each day from the litter box of a domestic cat. Prior to use, the sand was kept at room temperature in a sealed plastic container for 3–5 h. For each exposure, 20 g of the sand was placed into a Petri dish covered with nylon mesh and positioned inside the home cage of rats subjected to predator scent stress (PS). Exposures were performed once daily, between 13:00–14:00, for 10 consecutive days, with each session lasting 15 min. Control animals were presented with sand moistened with clean water. To prevent cross-contamination, PS and control groups were housed in separate rooms with independent air conditioning systems. Upon completion of the final exposure, animals underwent a 21-day recovery period before subsequent testing.

### 2.3. Drug Administration

The selective TAAR1 agonist LK00764 (ChemKonstant, Saint Petersburg, Russia; EC_50_ = 4.0 nM) was used for pharmacological intervention. This compound has previously demonstrated robust activity in several in vivo assays relevant to schizophrenia, including the attenuation of MK-801-induced hyperlocomotion, modulation of spontaneous activity in rats, behavioral effects in dopamine transporter knockout (DAT-KO) animals, and a reduction in stress-induced hyperthermia following intraperitoneal administration [39]. In the present study, LK00764 was administered intraperitoneally at a dose of 5 mg/kg once daily, beginning on day 15 of the experimental paradigm and continuing for five consecutive days. Control animals were treated with the corresponding vehicle solution under identical conditions.

The TAAR1 agonist LK00764 (EC_50_ = 4.0 nM) was administered at 5 mg/kg, i.p., once daily for 5 consecutive days. This regimen was selected based on the compound’s high potency and receptor selectivity, preclinical efficacy in models of psychiatric-relevant behaviors, and pharmacokinetic/pharmacodynamic properties. Once-daily dosing ensured sustained TAAR1 activation, while the 5-day course allowed robust behavioral and molecular effects to be observed without overt toxicity [39].

### 2.4. Experimental Protocol

**Figure 1:**
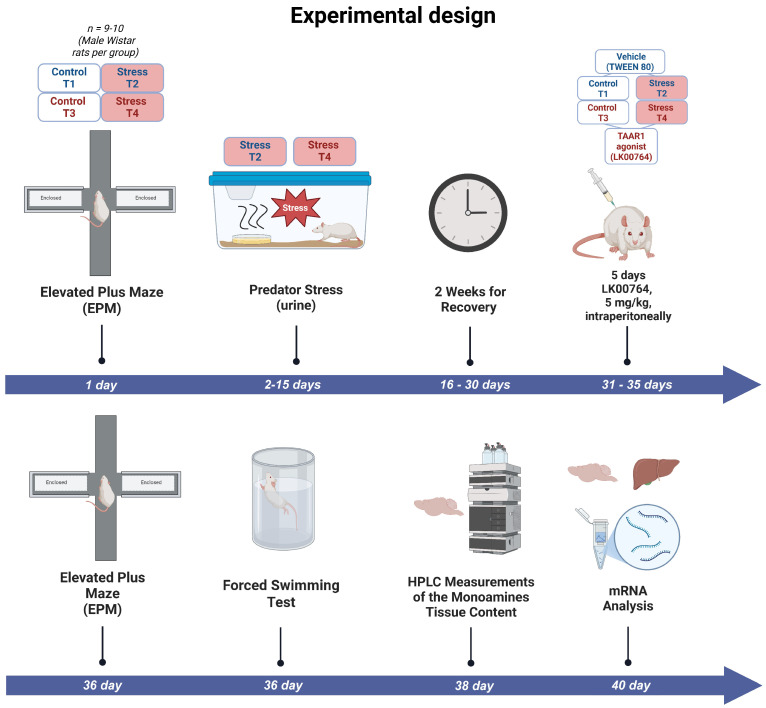
Experimental timeline outlining the sequence of predator stress exposures, TAAR1 agonist treatments, and behavioral assessments in the complex PTSD rat model. T1 = control; T2 = PS; T3 = LK00764, T4 = PS+LK00764.

Rats were randomly allocated to four experimental groups: (1) control animals receiving vehicle for 10 days (control, *n* = 9); (2) unstressed rats treated with LK00764 (LK00764, *n* = 8); (3) subjects exposed to repeated predator scent stress only (PS, *n* = 6); and (4) animals subjected to the PS paradigm that received TAAR1 agonist LK00764 (PS+LK00764, *n* = 7). All treatments, exposures, and behavioral assessments were conducted according to the timeline specified in the study design. The overall sequence of experimental procedures is illustrated in Figure 1.

### 2.5. Behavioral Tests

Anxiety-related responses were assessed using the elevated plus maze (EPM) based on the aversion of rats to open elevated spaces and their preference for enclosed arms [40]. Prior to testing, subjects acclimated to the behavioral suite before being placed at the intersection of the maze. For 5 min, spontaneous exploration within open and closed arms was continuously recorded and analyzed using a computerized video-tracking system (EthoVision XT 11.5, Noldus, Wageningen, The Netherlands). The relative time spent in each zone was calculated as the percentage of total experimental time spent in the open, closed, or center of the arena. Inter-trial cleaning of the apparatus with 3% hydrogen peroxide minimized olfactory interference between subjects. Specific behavioral parameters, such as rearing and duration spent in closed arms, were manually quantified from video recordings.

To confirm the development of a stress-responsive phenotype, the Porsolt forced swim test was implemented as described previously [41]. After a period of acclimatization to the test environment (40 min), each male rat was gently placed into a transparent cylinder (45 cm in height, 28 cm in diameter) filled with water maintained at 23 ± 1 °C. Over a 6-min observation period, two behavioral states—swimming and immobility—were evaluated, with the latency to initial immobility carefully recorded. The relative time spent in each behavioral state—active swimming, passive swimming (floating), diving, and climbing—was calculated as the percentage of total observation time during the 6-min session. This behavioral assay was conducted on the 36th day from the start of the experimental protocol, three weeks after cessation of the stress exposure.

### 2.6. Tissue Collection and Storage

Following completion of behavioral testing, rats were euthanized by an overdose of diethyl ether and immediately decapitated. Blood samples were collected ante-mortem for subsequent analyses. During necropsy, additional specimens including liver tissue were harvested. Brains were rapidly excised, and specific regions—the hippocampus and striatum—were dissected on ice in accordance with anatomical coordinates defined by the Paxinos and Watson atlas [42]. Tissue samples for qPCR and HPLC were snap-frozen in liquid nitrogen and stored at −70 °C until further biochemical assays, which were conducted within seven days of collection.

### 2.7. Quantification of Brain Monoamines and Their Metabolites

Twenty-four hours post behavioral assessment, rats were sacrificed by decapitation and the brains promptly removed. The hippocampus and striatum were carefully isolated from fresh brain tissue chilled on ice, guided by the Paxinos and Watson atlas [42], and immediately frozen using liquid nitrogen. Within seven days, monoamine levels were quantified. Brain samples were homogenized in 0.1 M perchloric acid, followed by centrifugation at 7000× *g* for 15 min at 4 °C. The resulting supernatants were passed through 0.2 µm syringe filters (Whatman, MI, USA) prior to analysis by high-performance liquid chromatography (HPLC) equipped with electrochemical detection. Chromatographic separation was accomplished on a Hypersil BDS C18 column (250 × 4.6 mm, 5 µm particle size) under isocratic conditions. The mobile phase consisted of 75 mM phosphate buffer (pH 4.6) containing 2 mM citric acid, 0.1 mM octanesulfonic acid, and 15% (*v*/*v*) acetonitrile. Electrochemical detection employed a glassy carbon working electrode set at +780 mV. Monoamine concentrations were expressed as picograms per milligram of tissue using external calibration curves [38].

### 2.8. Measurement of mRNA Expression Levels

#### 2.8.1. RNA Extraction

Total RNA was isolated from hippocampal tissue utilizing TRIzol reagent (Invitrogen, Oxford, UK), following the manufacturer’s guidelines. RNA yield and purity were determined spectrophotometrically using a NanoDrop 2000 instrument (Thermo Fisher Scientific, Waltham, MA, USA), with acceptable samples having an A_260_/A_280_ ratio above 1.8. RNA integrity was confirmed by evaluating the 18S and 28S ribosomal RNA bands through electrophoresis on a 1.4% agarose gel.

#### 2.8.2. cDNA Synthesis and Quantitative Real-Time PCR

Complementary DNA (cDNA) synthesis was performed using 2 μg of total RNA and the High-Capacity cDNA Reverse Transcription Kit (Applied Biosystems, Thermo Fisher Scientific, Waltham, MA, USA). Quantitative real-time PCR amplification was conducted with the Evrogen 5× qPCR Mix-HS SYBR Green (Evrogen, Moscow, Russia). Primer pairs were designed via Primer-BLAST and Primer3 Plus tools (NCBI, Boston, MA, USA). The sequences of primers applied in this study are presented in Table 1. Housekeeping genes peptidylprolyl isomerase A (PPIA) and glyceraldehyde 3-phosphate dehydrogenase (GAPDH) served as internal controls for normalization.

### 2.9. Gene Network Reconstruction

The reconstruction of gene networks is a critical approach to unraveling the complex molecular genetic interactions underlying biological processes and disease states [43]. In this context, we employed the ANDSystem, a cognitive computational platform developed at the Institute of Cytology and Genetics SB RAS, which leverages artificial intelligence-driven text mining to systematically extract relational data from scientific publications and biological databases [44]. This enables the comprehensive analysis of associative molecular networks encompassing genes, proteins, metabolites, microRNAs, and their regulatory and functional relationships with diseases, biological pathways, and phenotypes.

For the purpose of this study, we specifically utilized the ANDVisio module of the ANDSystem to reconstruct the gene network associated with TAAR1 signaling. The ANDVisio tool facilitates automated extraction and visualization of interactions among molecular entities, supporting integrative interpretation of genomic, proteomic, and metabolomic data. By mining a vast corpus of biomedical literature and curated databases, ANDSystem identifies molecular genetic objects and their interactions, including associative and regulatory links, thereby offering a robust framework to elucidate the gene–protein networks implicated in CPTSD pathophysiology and TAAR1-related neurobiological pathways.

### 2.10. Statistical Analysis

All statistical analyses were performed in Python 3.12, utilizing the libraries numpy, pandas, scipy, statsmodels, matplotlib, and scikit-posthocs for data handling, statistical modeling, and visualization. Data import, preprocessing, and extraction of numeric variables were managed using pandas.

Normality of distributions was evaluated with the Shapiro–Wilk test, and homogeneity of variances assessed via Levene’s test (scipy.stats). For each variable, if parametric assumptions were met for all groups, a one-way ANOVA (statsmodels) was computed; otherwise, the non-parametric Kruskal–Wallis test was applied. Effect sizes were quantified as eta squared ($\eta^{2}$) for ANOVA and $\eta_{KW}^{2}$ for Kruskal–Wallis. Statistical power was estimated from effect size using the FTestAnovaPower function in statsmodels. For variables demonstrating significant main effects, Tukey’s HSD or Dunn’s test (with Bonferroni correction) was used for post hoc pairwise comparisons, as appropriate.

In addition, two-way ANOVA was performed for variables with coded factors, incorporating interaction terms if normality and homoscedasticity were satisfied. Effect sizes and power were calculated as above.

Graphical summaries included effect size barplots for one-way, two-way, and Kruskal–Wallis tests, generated in matplotlib. Result tables and summary figures were programmatically exported as CSV and PNG files, respectively. All statistical tests were two-sided, with a significance threshold of α=0.05, and *p*-values were annotated by a standardized asterisk scheme generated by a custom Python function: *** (p<0.001), ** (p<0.01), * (p<0.05), or ns (not significant, p≥0.05).

## 3. Results

### 3.1. Data Consistency and Final Group Sizes

Prior to the onset of the experimental procedures, forty male rats were initially enrolled and screened for baseline behavioral characteristics. The intended experimental design comprised four groups (control, LK00764, PS, and PS+LK00764) with ten animals per group. However, pre-experimental behavioral assessment revealed that ten animals exhibited markedly elevated anxiety-related activity, indicative of a pre-existing anxious phenotype. To avoid potential confounding effects on stress reactivity and treatment outcomes, these animals were excluded from further participation (Figure S1).

As a result, thirty rats were distributed across the four experimental groups in the following allocation: control (n=9), LK00764 (n=8), PS (n=6), and PS+LK00764 (n=7). Prior to experimental manipulations, all animals were housed in eight home cages (five animals per cage) under standard laboratory conditions.

Several samples were excluded from the biochemical analysis of neurotransmitter and metabolite levels in the striatum and hippocampus due to technical issues encountered during measurement, which prevented reliable quantification. In addition, for one sample from the PS group in which D2R expression was measured, the recorded value exceeded the group mean by more than an order of magnitude and was therefore excluded from the final analysis as an extreme outlier.

This adjusted allocation forms the basis for the final dataset and accounts for the group size variability reported in the CONSORT-style summary table (Table 2).

### 3.2. TAAR1 Agonist Significantly Ameliorates Behavioral Deficits in Predator Scent-Stressed Rats

Table 3 and Figures S2–S5 summarize the impact of the selective TAAR1 agonist LK00764 on anxiety-related behaviors observed in rats exposed to predator scent stress. One-way ANOVA revealed pronounced differences in behavioral responses among experimental groups (Table S1). Significant effects were observed for active and passive swimming behaviors, while diving and climbing remained unchanged (Table S2). Specifically, active swimming showed a strong group effect ($F_{3,26}=13.40$, p<0.001, $\eta^{2}=0.607$), indicating substantial modulation of behavioral activation. Passive swimming (immobility) also differed significantly among groups ($F_{3,26}=5.99$, p=0.003, $\eta^{2}=0.409$), reflecting alterations in behavioral despair associated with stress exposure and treatment effects. In contrast, the number of dives ($F_{3,26}=0.59$, p=0.628, $\eta^{2}=0.064$) and climbing activity ($F_{3,26}=0.48$, p=0.698, $\eta^{2}=0.053$) did not differ significantly, suggesting that these specific motor components were not sensitive to experimental manipulation. The treatment primarily shifted the balance between active and passive coping in the forced swim test, with minimal effects on general locomotion. This pattern supports a stress-related pharmacological mechanism.

A two-way ANOVA was performed to evaluate the main effects of treatment with LK00764, PS, and their interaction across multiple behavioral and biochemical brain parameters. The analysis considered three factors per variable: C(LK00764), C(PS), and the interaction C(LK00764):C(PS). Table S3 summarizes the *F*-values, degrees of freedom, *p*-values, and effect sizes ($\eta^{2}$), respectively.

No significant main effect of LK00764 or PS was observed for most parameters (all p>0.3), except for a subset where highly significant effects emerged. Notably, for active swimming, a robust effect of LK00764 was found ($F_{\left(1,26\right)}=18.45$, p<0.001, $\eta^{2}=0.42$, Figure S4), along with a strong effect of PS ($F_{\left(1,26\right)}=24.31$, p<0.001, $\eta^{2}=0.48$). Both factors explained large proportions of variance ($\eta^{2}>0.25$). For the interaction C(LK00764):C(PS), no significant effects were detected across all datasets (p>0.43), indicating the absence of synergistic or antagonistic interactions between treatments on the measured outcomes.

For passive swimming, significant main effects of both LK00764 ($F_{\left(1,26\right)}=9.54$, p=0.0047, $\eta^{2}=0.27$), and PS ($F_{\left(1,26\right)}=9.07$, p<0.001, $\eta^{2}=0.26$), were observed, again indicating pronounced independent contributions of both treatments to phenotypic variability. For measures or behavioral scores with non-significant results (p>0.3, F<1.1), effect sizes ($\eta^{2}$) remained low (<0.03), and post hoc power analysis indicated inadequate statistical power to reliably detect true effects (power < 0.6).

Normality (Shapiro–Wilk *p*) and homoscedasticity (Levene’s test *p*) assumptions were supported for all measured parameters (p>0.16 for normality, p>0.51 for variance), confirming suitability of parametric analysis.

Analysis of exploratory behavior using the elevated plus maze revealed significant treatment effects for the time spent in the closed and open arms, whereas the time spent in the center of the maze did not differ significantly among groups (Tables S4 and S5). The Kruskal–Wallis test for time spent in closed arms was significant (H=15.65, p=0.001, $\eta^{2}=0.54$), indicating a strong effect of treatment. Post hoc Dunn’s test revealed that control animals spent significantly less time in closed arms compared to the PS (p=0.010) and PS+LK00764 (p=0.029) groups, while no other pairwise comparisons reached significance. These findings suggest that PS treatment induced an anxiogenic effect, reflected by an increased preference for the closed arms, whereas co-administration of LK00764 did not further modify this response.

Similarly, a significant treatment effect was observed for time spent in open arms (H=19.98, p<0.001, $\eta^{2}=0.69$). Dunn’s post hoc analysis showed that PS-treated animals spent significantly less time in the open arms compared to both the LK00764 (p<0.001) and control (p=0.028) groups, further supporting the anxiogenic profile of PS. In contrast, the time spent in the center zone did not differ significantly among experimental groups (H=7.10, p=0.069, $\eta^{2}=0.24$), suggesting that the treatments did not substantially affect general locomotor activity. Altogether, these results indicate that PS administration elicits anxiety-like behavior in the elevated plus maze, while LK00764 may exert a partial but insufficient modulatory effect on this response.

### 3.3. Effect of TAAR1 Agonist on Monoamine and Metabolite Levels in the Striatum

**Figure 2:**
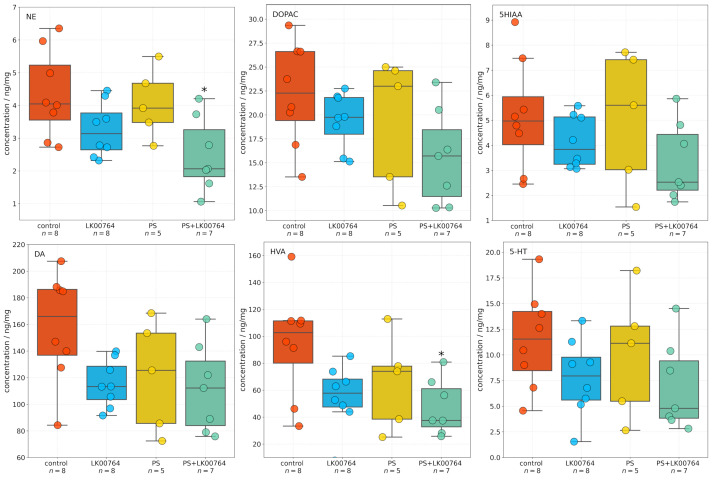
Effects of LK00764 treatment on striatal neurotransmitter and metabolite concentrations in four experimental groups (control, LK00764, PS, PS+LK00764) shown as boxplots. Statistically significant differences between groups are indicated as * = effect compared to control group; * *p* < 0.05. NE = norepinephrine, DOPAC = 3,4-dihydroxyphenylacetic acid, 5HIAA = 5-hydroxyindoleacetic acid, DA = dopamine, HVA = homovanillic acid, 5-HT = serotonin.

One-way ANOVA (Figure S6) revealed significant effects of treatment type on NE, DA, and homovanillic acid (HVA) concentrations in the striatum (all p<0.05), whereas 3,4-dihydroxyphenylacetic acid (DOPAC) 5-hydroxyindoleacetic acid (5HIAA) and 5-HT levels were not significantly altered (Figure 2). Effect sizes, estimated using eta squared ($\eta^{2}$), indicated moderate to large effects for NE ($\eta^{2}=0.34$), DOPAC ($\eta^{2}=0.22$), DA ($\eta^{2}=0.28$), and HVA ($\eta^{2}=0.31$), suggesting that a substantial proportion of the variance in neurotransmitter concentrations was attributable to the experimental manipulation.

Post hoc Tukey multiple comparisons were conducted to further examine group differences in striatal neurotransmitter and metabolite concentrations following stress exposure and treatment. The analysis revealed statistically significant differences for several compounds, predominantly involving the PS+LK00764 and control groups. For NE, a significant increase was observed in the PS+LK00764 group compared to controls ($p_{adj}=0.018$), indicating a specific effect of combined stress and LK00764 treatment on noradrenergic activity in the striatum. No other pairwise comparisons for NE reached statistical significance, although a trend was observed between the PS and PS+LK00764 groups ($p_{adj}=0.100$).

HVA concentrations were also significantly higher in the PS+LK00764 group relative to controls ($p_{adj}=0.029$), further supporting enhanced dopaminergic turnover under the combined treatment condition. No other comparisons for HVA were significant.

For 5HIAA DOPAC, DA, and 5-HT, no statistically significant differences were detected across groups, indicating relative stability of serotonergic metabolism in the striatum under the experimental conditions tested.

Overall, these post hoc analyses highlight a consistent pattern whereby the combination of stress exposure and LK00764 treatment (PS+LK00764) significantly alters noradrenergic and dopaminergic neurotransmission in the striatum, as evidenced by increases in NE and HVA. These results provide robust evidence for a modulatory effect of LK00764 under conditions of stress, potentially implicating this compound in the regulation of striatal neurochemical pathways relevant to stress-related neuropathology.

Full statistical details, including all pairwise Tukey HSD comparisons, are provided in Tables S6 and S7 in the Supporting Information. These results collectively underscore that combined stress and pharmacological intervention produce measurable alterations in striatal neurochemistry, whereas serotonergic markers remained largely unaffected.

Two-way ANOVA (Table S8, Figure S7) was performed to investigate the effects of LK00764 treatment and predator stress exposure on striatal neurotransmitter and metabolite concentrations. Results indicated significant main effects of treatment for NE ($F_{\left(1,24\right)}=9.39$, p=0.005, $\eta^{2}=0.266$), DA ($F_{\left(1,24\right)}=4.91$, p=0.036, $\eta^{2}=0.150$), and HVA ($F_{\left(1,24\right)}=6.97$, p=0.014, $\eta^{2}=0.205$), indicating robust modulatory effects of LK00764 administration on noradrenergic and dopaminergic neurochemistry in the striatum. No statistically significant effects were found for 5HIAA or 5-HT, indicating that serotonergic metabolism remained relatively stable under the experimental conditions. Furthermore, none of the interaction terms between treatment and stress exposure reached statistical significance across compounds, suggesting that LK00764’s influence on striatal neurochemistry is largely independent of stress exposure.

Effect size estimates ($\eta^{2}$) revealed moderate to large effects for NE, DA, and HVA, indicating that a substantial proportion of variance in these neurotransmitter systems can be attributed to LK00764 treatment. These findings collectively suggest that LK00764 selectively modulates dopaminergic and noradrenergic neurotransmission in the striatum, with no significant interaction with stress exposure, highlighting its potential therapeutic role in stress-related neuropathology.

### 3.4. Effect of TAAR1 Agonist on Monoamine and Metabolite Levels in the Hippocampus of CPTSD Rats

**Figure 3:**
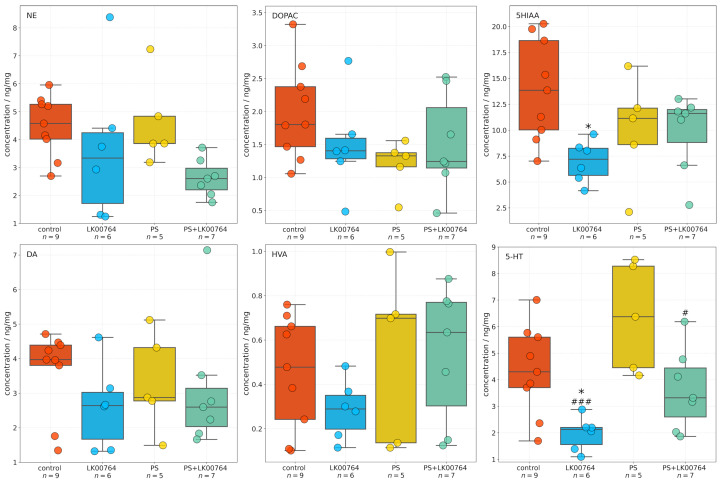
Boxplots showing the effects of LK00764 treatment on hippocampal neurotransmitter and metabolite concentrations across four experimental groups (control, LK00764, PS, and PS+LK00764). Statistically significant differences between groups are indicated as * = effect compared to control group; ^#^ = effect compared to PS group; * *p* < 0.05, ^#^ *p* < 0.05, ^###^ *p* < 0.001. NE = norepinephrine, DOPAC = 3,4-dihydroxyphenylacetic acid, 5HIAA = 5-hydroxyindoleacetic acid, DA = dopamine, HVA = homovanillic acid, 5-HT = serotonin.

High-performance liquid chromatography was used to quantify neurotransmitter and metabolite concentrations in the hippocampus across four experimental groups. One-way ANOVA and Kruskal–Wallis (Table S9, Figure S8) in the hippocampus revealed a significant group effect for 5-HT ($F_{\left(3,23\right)}=7.53$, p<0.001, $\eta^{2}=0.50$) and 5HIAA ($F_{\left(3,23\right)}=8.31$, p=0.040, $\eta^{2}=0.32$), indicating a large effect size (Figure 3). This finding suggests a robust modulation of hippocampal serotonergic activity in response to treatment and stress exposure. In contrast, no statistically significant group effects were observed for NE($F_{\left(3,23\right)}=2.30$, p=0.104, $\eta^{2}=0.23$), DA(H=2.90, p=0.408, $\eta^{2}=0.11$), DOPAC($F_{\left(3,23\right)}=1.64$, p=0.207, $\eta^{2}=0.18$), or HVA($F_{\left(3,23\right)}=1.09$, p=0.375, $\eta^{2}=0.12$). Although these effects did not reach statistical significance, their medium $\eta^{2}$ values suggest potential treatment-related trends that may become significant with larger sample sizes (Figure S8). The observed pattern points to a preferential involvement of serotonergic mechanisms in the hippocampal response to treatment, with dopaminergic and noradrenergic parameters showing only suggestive, non-significant changes.

Post hoc analyses employing Tukey’s or Dunn’s test were performed following omnibus testing to evaluate intergroup differences for each compound (see Table S10). None of the pairwise comparisons for NE, DOPAC, HVA, or DA reached statistical significance following adjustment for multiple comparisons ($p_{adj}>0.1$ for all contrasts). Although mean differences were observed between some groups (e.g., PS+LK00764 vs. PS for NE, meandiff =1.965), the associated adjusted *p*-values indicated no statistically reliable effects (all $p_{adj}>0.05$).

For hippocampal 5-HT, a significant decrease was detected in the LK00764 group relative to the PS group (Dunn’s test, $p_{adj}<0.001$) and control group (Dunn’s test, $p_{adj}=0.037$), indicating treatment-specific modulation of serotonergic tone. A significant difference was also observed between the PS+LK00764 and PS groups ($p_{adj}=0.031$). All other pairwise contrasts for 5-HT were non-significant following correction for multiple comparisons ($p_{adj}>0.12$). Additionally, hippocampal 5HIAA levels were significantly lower in the LK00764 group compared to the control group ($p_{adj}=0.024$), further supporting the presence of treatment-related alterations in serotonergic metabolism.

Taken together, these findings demonstrate that LK00764 administration in the context of psychosocial stress produces a specific effect on hippocampal 5-HT and 5HIAA levels, while levels of other monoamines and their metabolites remain unchanged among experimental groups.

### 3.5. Effect of TAAR1 Agonist on mRNA Expression of SERT, 5-HT3A, MAO-A, MAO-B, COMT, and BDNF in the Striatum of CPTSD Rats

Baseline gene expression levels in the control group approximated unity, consistent with normalized fold-change values. Expression levels of BDNF (0.934 ± 0.013), dopamine D2 receptor (D2R; 1.035 ± 0.061), and monoamine oxidase B (MAO-B; 1.026 ± 0.057) were slightly elevated relative to the normalization standard.

**Figure 4:**
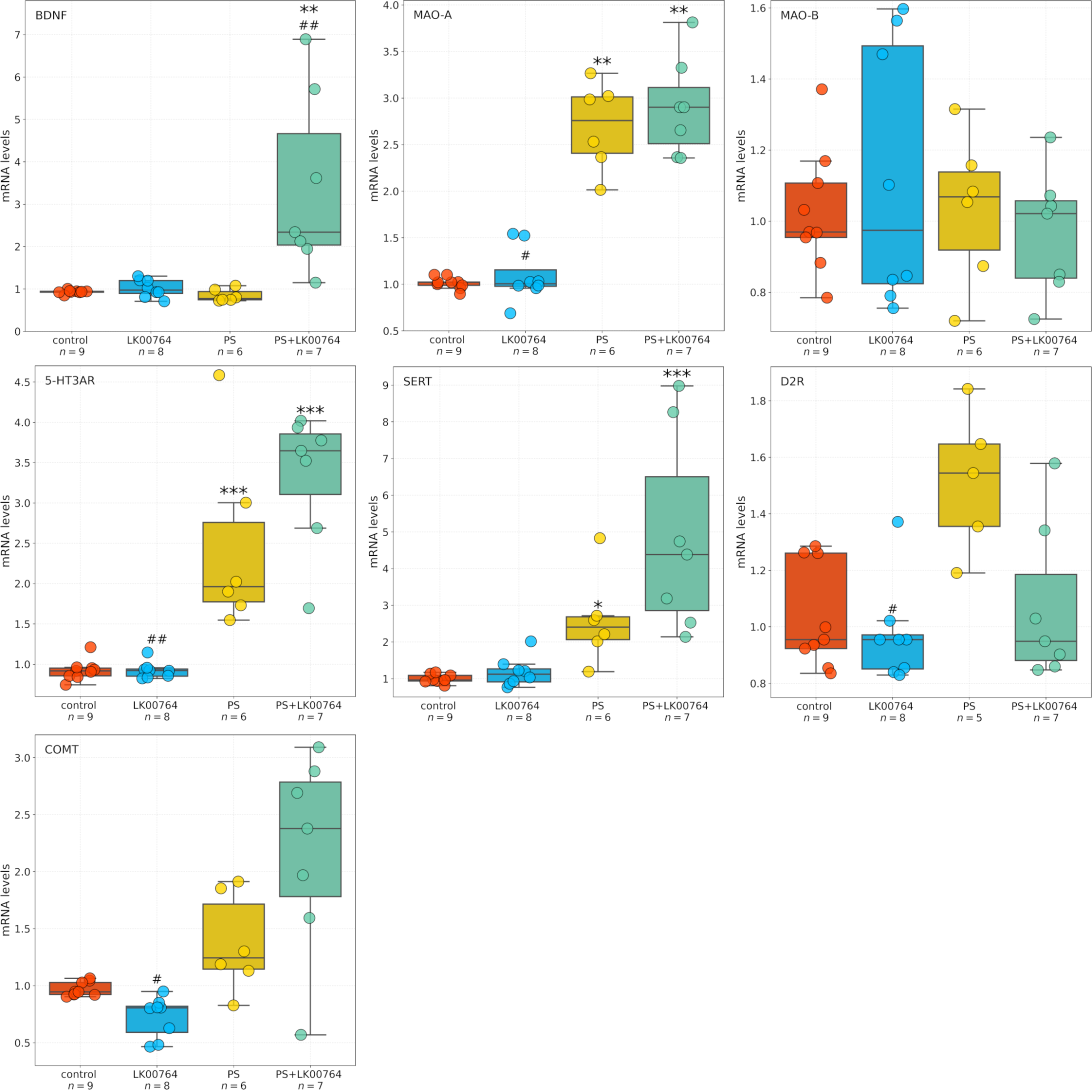
Effects of LK00764 treatment on striatal gene expression in a rat model of CPTSD. Boxplots illustrate the distribution of mRNA expression levels for SERT, 5-HT3A, MAO-A, MAO-B, COMT, and BDNF across experimental groups. * = effect compared to control group; ^#^ = effect compared to PS group; * *p* < 0.05; ** *p* < 0.01; *** *p* < 0.001; ^#^ *p* < 0.05; ^##^ *p* < 0.01.

Figure 4 shows box plots depicting the mRNA expression levels of neurotransmission-related genes in the striatum. To assess the differential expression of key serotonergic and dopaminergic genes implicated in monoaminergic neurotransmission and neuroplasticity in the striatum of CPTSD rats, a Kruskal–Wallis non-parametric analysis was performed for each transcript (Table S11, Figure S9).

Among the genes analyzed, all targets—except MAO-B—showed statistically significant group differences in striatal expression, as revealed by ANOVA and Kruskal–Wallis tests. BDNF displayed a robust modulation (H=16.06, p=0.001, $\eta_{\mathrm{KW}}^{2}$ = 0.55), indicating substantial alterations in neurotrophic signaling within the striatum of CPTSD rats. Similarly, D2R expression showed a significant difference across experimental groups (H=9.04, p=0.029), suggesting dopaminergic dysregulation associated with CPTSD-related phenotypes.

MAO-A expression showed the most pronounced effect (H=21.42, p<0.001), corresponding to a large effect size derived from the nonparametric test ($\eta_{\mathrm{KW}}^{2}$ = 0.74), (Figure S9), consistent with a strong deviation among groups and implying substantial changes in monoamine oxidative metabolism.

Following the Kruskal–Wallis analysis, Dunn’s post hoc test with Bonferroni correction was performed to delineate specific pairwise group differences (Table S12). Significant effects ($p_{adj}<0.05$) were detected across several genes, highlighting distinct stress- and drug-related transcriptional patterns in the striatum of CPTSD rats.

For BDNF, multiple significant pairwise differences were observed between groups. The combined stress and drug condition (PS+LK00764) differed significantly from the drug-only group (LK00764; $p_{adj}=0.048$), from the stress-only group (PS; $p_{adj}=0.001$), and from controls ($p_{adj}=0.009$). These results indicate that the interaction between stress exposure and LK00764 treatment profoundly modifies BDNF transcription, producing a distinct molecular phenotype not observed under either condition alone. In line with the large Kruskal–Wallis effect size ($\eta_{KW}^{2}$ = 0.55), these differences suggest that combined stress and pharmacological intervention exert a synergistic effect on neurotrophic signaling within the striatum.

D2R expression also exhibited significant modulation, with notable differences between the drug-only group and the stress-only group (LK00764 vs. PS; $p_{adj}=0.037$). This pattern reflects a robust dopaminergic response to stress and suggests that LK00764 may partially reverse or counteract stress-induced alterations in dopaminergic receptor expression.

For MAO-A, strong and consistent effects were found across multiple pairwise comparisons. The drug-only group differed significantly from both PS ($p_{adj}=0.015$) and PS+LK00764 ($p_{adj}=0.006$) groups, while both PS ($p_{adj}=0.008$) and PS+LK00764 ($p_{adj}=0.003$) differed from controls. These findings indicate pronounced stress-related upregulation of monoamine oxidase A, which is further modulated by drug administration. The combined pattern suggests that stress exposure drives substantial enzymatic changes in monoaminergic metabolism, with LK00764 exerting an additional, potentially bidirectional regulatory influence.

In the case of COMT, significant differences were detected between LK00764 and PS ($p_{adj}=0.023$) as well as between LK00764 and PS+LK00764 ($p_{adj}<0.001$). These results indicate that pharmacological modulation of catechol-*O*-methyltransferase occurs primarily in the absence of stress, and that the presence of stress fundamentally alters the drug’s effect on COMT transcription. This pattern is consistent with a context-dependent pharmacodynamic profile.

MAO-B expression did not differ significantly across any pairwise comparisons ($p_{adj}=1$ for all pairs), suggesting that this isoform remains stable under both stress and pharmacological conditions, in contrast to the pronounced changes observed for MAO-A.

Significant post hoc differences were also observed for the 5-HT3A receptor. The drug-only group differed from both PS ($p_{adj}=0.001$) and PS+LK00764 ($p_{adj}<0.001$), while stress-only and combined groups differed significantly from controls (PS vs. control, $p_{adj}<0.001$; PS+LK00764 vs. control, $p_{adj}<0.001$). This pattern parallels that observed for MAO-A and supports the involvement of serotonergic receptor systems in stress-induced neuroplastic adaptations and their pharmacological modulation by LK00764.

For SERT, significant pairwise differences were found between LK00764 and PS+LK00764 ($p_{adj}=0.007$), between PS and control ($p_{adj}=0.024$), and between PS+LK00764 and control ($p_{adj}<0.001$). These results suggest a strong stress-related increase in 5-HT transporter expression, further modified by combined stress–drug exposure, indicating that the drug’s effects on serotonergic regulation are contingent upon the stress context.

Collectively, the Dunn’s post hoc analysis confirms that stress robustly alters the transcription of multiple monoaminergic and neurotrophic genes in the striatum, including D2R, MAO-A, 5-HT3A, and SERT, while leaving MAO-B unaffected. The combined stress–drug condition (PS+LK00764) consistently produced unique transcriptional signatures, emphasizing the context-dependent nature of pharmacological modulation. The observed large $\eta_{KW}^{2}$ values for several genes (e.g., BDNF, MAO-A, D2R) further underscore the magnitude of these effects. Together, these findings suggest that striatal monoaminergic and neurotrophic systems undergo complex, gene-specific remodeling in response to stress and its pharmacological modulation, highlighting potential molecular substrates of stress resilience and treatment response in CPTSD.

### 3.6. Effect of TAAR1 Agonist on mRNA Expression of SERT, 5-HT3A, MAO-A, MAO-B, COMT, and BDNF in the Hippocampus of CPTSD Rats

**Figure 5:**
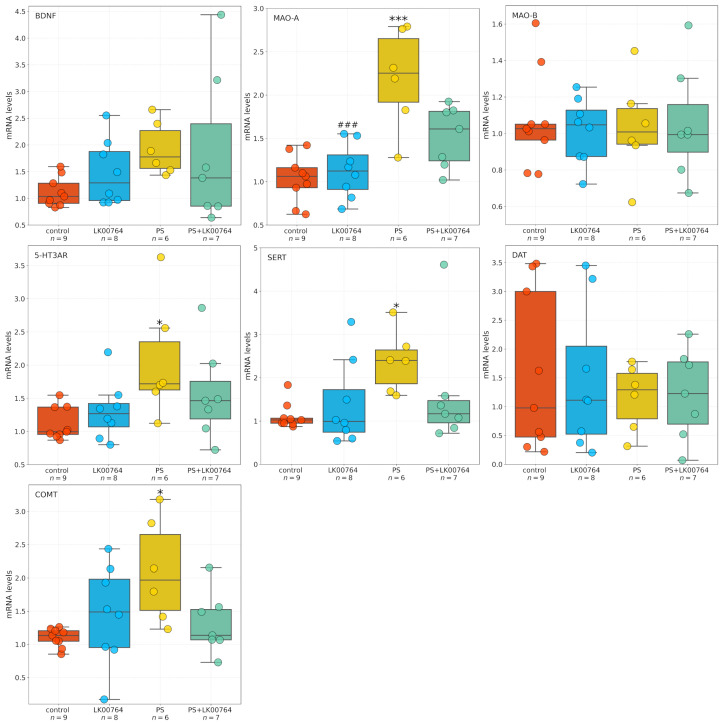
Effects of LK00764 treatment on hippocampal gene expression in a CPTSD rat model. Boxplots represent the distribution of mRNA expression levels for SERT, 5-HT3A, MAO-A, MAO-B, COMT, and BDNF across experimental groups. * = effect compared to control group; ^#^ = effect compared to PS group; * *p* < 0.05; *** *p* < 0.001; ^###^ *p* < 0.001.

The effects of the TAAR1 agonist on mRNA expression of SERT, 5-HT3A, MAO-A, MAO-B, COMT, and BDNF in the hippocampus of CPTSD rats (Figure 5) were analyzed using one-way ANOVA or Kruskal-Wallis tests, depending on data distribution (Table S13).

For BDNF, ANOVA test revealed no significant differences between groups ($p_{adj}=0.200$, $\eta_{KW}^{2}=0.16$). Similarly, COMT expression did not differ significantly among groups ($p_{adj}=0.045$, $\eta_{KW}^{2}=0.28$), although a trend toward significance was observed. In contrast, SERT expression showed a significant effect of the TAAR1 agonist treatment ($p_{adj}=0.034$, $\eta_{KW}^{2}=0.30$), indicating a moderate effect size. Likewise, 5-HT3A expression was significantly altered ($p_{adj}=0.035$, $\eta_{KW}^{2}=0.30$), representing the largest effect among the genes tested. DAT mRNA expression did not differ between groups ($p_{adj}=1.00$, $\eta_{KW}^{2}<0.001$), indicating no effect of treatment.

For MAO-A, a significant main effect was detected by ANOVA ($F_{\left(3,26\right)}=13.281$, p<0.001, $\eta^{2}=0.61$), indicating a very large effect size. Post hoc comparisons (Table S14) confirmed substantial group differences. Conversely, MAO-B expression showed no significant group differences ($F_{\left(3,26\right)}=0.081$, p=0.970, $\eta^{2}=0.01$), indicating no detectable effect of the TAAR1 agonist.

Statistical analysis revealed differential effects of TAAR1 agonist treatment (LK00764) on the mRNA expression of selected genes implicated in monoaminergic signaling and neuroplasticity within the hippocampus. Comparisons were performed using Dunn’s post hoc test for non-parametric data and Tukey HSD for parametric data (Table S14). For BDNF, no statistically significant differences in mRNA expression were observed between groups (all adjusted p>0.05). A trend toward reduced expression was noted in the PS group compared to controls (p=0.247), though this did not reach statistical significance.

Analysis of SERT revealed a significant increase in hippocampal mRNA levels in the PS group compared to controls (p=0.041), whereas no other group comparisons reached significance. COMT expression was significantly reduced in PS-exposed rats relative to controls (p=0.029, Dunn’s test), whereas no significant differences were observed in other group comparisons. For 5-HT3A, Dunn’s test revealed a significant decrease in mRNA expression in the PS group compared to controls (p=0.025), with no significant differences observed between other groups. No statistically significant differences in DAT expression were detected among groups (all p>0.05).

A robust effect of TAAR1 agonist treatment was observed on MAO-A mRNA expression. PS exposure significantly increased MAO-A expression relative to LK00764-treated rats (p<0.001) and controls (p<0.001). Co-administration of the TAAR1 agonist (PS+LK00764) significantly attenuated this upregulation compared to PS alone (p=0.015), restoring MAO-A levels toward baseline. Nevertheless, PS+LK00764 expression remained significantly different from controls (p=0.044), indicating partial normalization. Tukey HSD analysis did not reveal significant differences in MAO-B expression among experimental groups (all p>0.05).

Overall, these results indicate that PS exposure induces significant upregulation of MAO-A and downregulation of COMT and 5-HT3A in the hippocampus, whereas TAAR1 agonist treatment selectively mitigates MAO-A overexpression. This suggests a potential modulatory role in stress-related monoaminergic dysregulation. No significant changes were observed for BDNF, SERT, or DAT expression.

### 3.7. Effect of TAAR1 Agonist on MAO-A Gene Expression in the Liver

**Figure 6:**
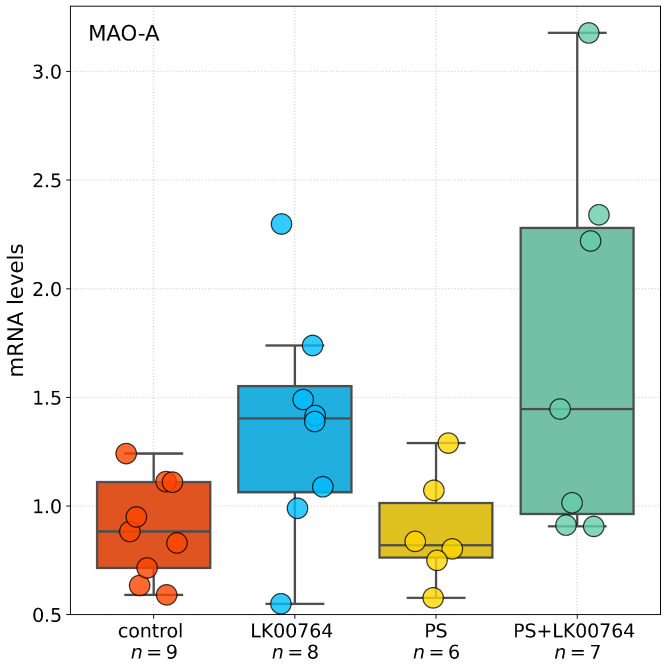
Boxplot representation of MAO-A gene expression in the liver across experimental groups. Boxes show the interquartile range, whiskers indicate data variability outside the upper and lower quartiles, and horizontal lines denote median values for each group.

Kruskal–Wallis analysis revealed a significant difference in MAO-A mRNA expression in the liver (Figure 6) across experimental groups (p=0.030, $\eta_{KW}^{2}=0.31$). However, Dunn’s post hoc comparisons (Table S15) revealed no statistically significant differences between groups (all adjusted p>0.05), with the closest to significance observed between the PS+LK00764 and control groups ($p_{adj}=0.142$). These results suggest that while PS exposure and TAAR1 agonist treatment may influence hepatic MAO-A expression, such effects are less robust compared to the hippocampus. This supports the notion of a tissue-specific modulation of MAO-A by stress and TAAR1 agonism.

### 3.8. Gene Network Reconstruction: Hub Analysis of CPTSD Pathways Linked with TAAR1

**Figure 7:**
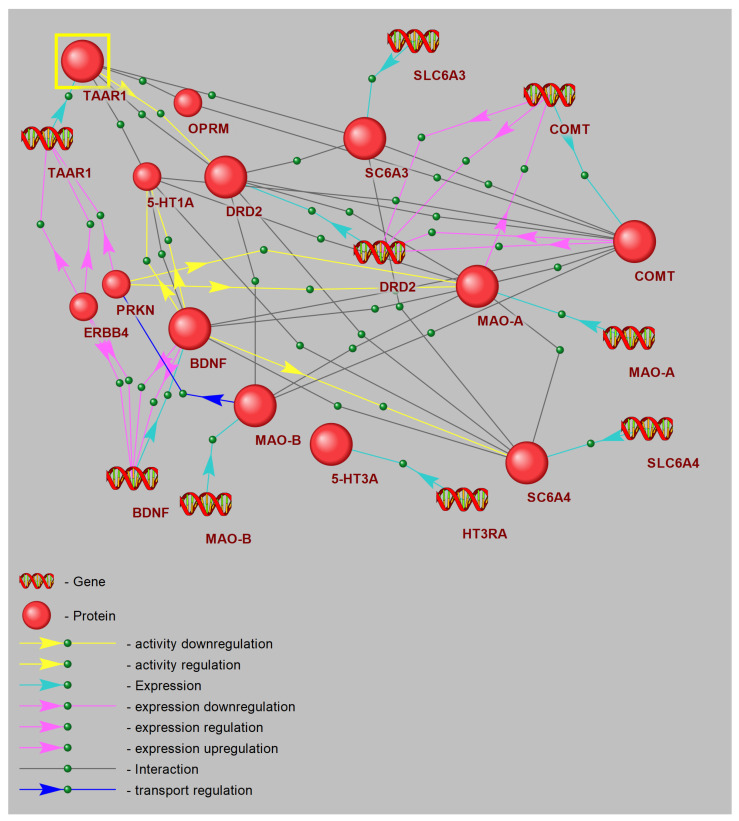
Schematic illustration of a node within the gene interaction network.

The reconstructed gene network diagram defines a core “trace amine” signaling hub centered on TAAR1, represented as the principal node in the upper left, highlighted for emphasis. TAAR1 directly engages regulatory connections with critical monoaminergic and neurotrophic genes involved in stress adaptation and mood control. Excitatory links extend from TAAR1 to genes encoding the dopamine D2 receptor (DRD2), serotonin 1A receptor (5-HT1A), and the dopamine transporter (SLC6A3), representing direct modulation of dopaminergic and serotonergic receptor and transporter activity (Figure 7).

Additionally, modulatory interactions are depicted by green dashed lines from TAAR1 to COMT and MAO-A, reflecting indirect regulation of catecholamine metabolic pathways. DRD2 emerges as a central hub, integrating input from TAAR1 and projecting onto downstream targets including COMT, MAO-A, and the 5-HT transporter gene (SLC6A4). This arrangement emphasizes the role of D2 signaling in orchestrating both dopamine clearance and 5-HT uptake.

On the right, COMT and MAO-A nodes are annotated with DNA icons, signifying key sites of transcriptional regulation. Bidirectional connections between COMT, DRD2, and SLC6A3 illustrate feedback mechanisms between receptor activity, transporter dynamics, and monoamine degradation. MAO-A further links to DRD2 and to the trace amine chemoreceptor gene, revealing crosstalk between trace amine signaling and oxidative deamination.

5-HT1A is mapped below TAAR1, feeding into the neurotrophin gene BDNF through excitatory projections, indicating that 5-HT receptor activation upregulates neurotrophin expression. BDNF subsequently acts as a central neuroplasticity modulator, sending strong effector signals to MAO-B and related mitochondrial monoamine oxidase pathways.

The network also incorporates SLC6A4 and SLC6A3, with connections to their neurotransmitter substrates, capturing reuptake interactions. PRKN (parkin) and ERBB4 form a parallel branch, establishing links from TAAR1 to BDNF via co-regulation of neuroprotective ubiquitin ligase and ErbB signaling influences on neurotrophin transcription. The μ-opioid receptor (OPRM) completes the diagram, positioned above DRD2 with minor connectivity back to TAAR1, reflecting potential cross-talk between opioid and trace amine systems.

Collectively, these results designate TAAR1 as a master regulatory node coordinating receptor activity, transporter gene expression, and metabolic enzyme levels, while integrating neurotrophic and ubiquitin-ligase functions to modulate synaptic plasticity and foster resilience to chronic stress.

## 4. Discussion

TAAR1 agonists such as ulotaront represent a novel and increasingly compelling pharmacological class, originally developed for schizophrenia but now drawing broader interest due to their modulatory effects on monoaminergic and stress-responsive systems. Ulotaront, a selective TAAR1 agonist with additional 5-HT_1A_ activity, has demonstrated robust efficacy in clinical trials for schizophrenia, showing improvements across symptom domains while exhibiting a markedly more favorable side-effect profile compared with conventional dopamine D_2_-targeting antipsychotics [45,46,47].

Although most clinical progress has focused on psychotic disorders, accumulating preclinical evidence indicates that TAAR1 activation also influences anxiety- and stress-related phenotypes. These observations raise the possibility that TAAR1 agonists could extend therapeutic benefit to stress-related psychopathologies, including CPTSD [33,34,48]. However, this potential has not yet been systematically tested in clinical populations.

Given the substantial unmet need for mechanistically novel treatments for PTSD, the emerging pharmacological and behavioral profile of TAAR1 agonists warrants dedicated investigation, particularly in light of their capacity to modulate neural circuits central to stress responsivity, emotional regulation, and monoaminergic homeostasis.

**Figure 8:**
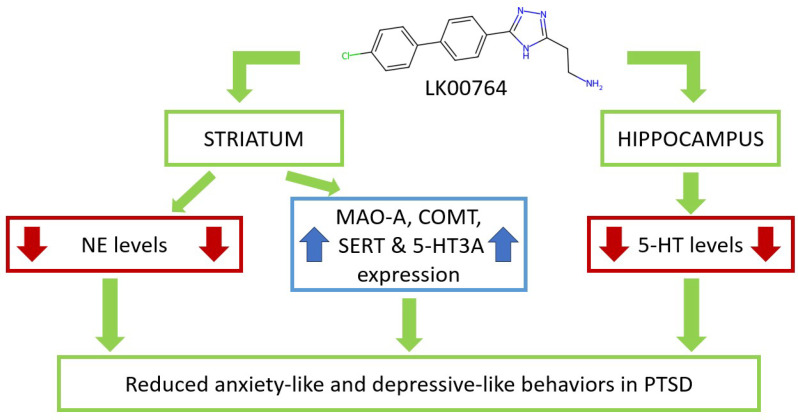
Schematic representation of LK00764 effects on striatal and hippocampal neurotransmitters, metabolites, and gene expression, modulating anxiety- and depressive-like behaviors in CPTSD.

Our findings are in strong agreement with previous studies demonstrating that TAAR1 agonists attenuate PTSD-like behavior (Figure 8) [34]. In contrast to these earlier reports, the present study provides the first evidence obtained using an animal model of CPTSD. The ability of TAAR1 agonists to mitigate both simple PTSD and CPTSD symptoms enhances their translational potential for future clinical trials. Importantly, CPTSD was associated not only with anxiety-related manifestations but also with depression-like behavior.

Anxiety-like behavior was confirmed using the EPM test and was characterized by an imbalance in the time spent in open versus closed arms and the central zone. In CPTSD rats, time spent in the open arms was markedly reduced, whereas time spent in the closed arms increased, leading to a significant elevation in the anxiety index. Notably, the present data were obtained three weeks after cessation of predator stress exposure, whereas our previous studies examined EPM performance only two weeks post-stress [4,5].

Increased immobility time in the Porsolt forced swim test (PST) in CPTSD rats suggests the presence of depression-like behavior [49,50,51,52]. Depression, although not part of the core CPTSD symptom cluster, is considered a frequent comorbid behavioral disorder [53,54]. Individuals with depression are more likely to experience traumatic exposure, while CPTSD itself can precipitate depressive episodes [55]. Indeed, approximately half of individuals diagnosed with PTSD either currently suffer from or have a history of major depressive episodes [56]. Clinically, antidepressants—particularly selective 5-HT reuptake inhibitors (SSRIs)—are widely used for pharmacological management of PTSD [57]. In our model, the strong positive correlation between time spent in closed arms and immobility time in the PST (r=0.83, p<0.05) indicates a synergistic relationship between anxiety- and depression-like behaviors.

Additional analyses of the elevated plus maze conducted before stress exposure and following treatment are provided in the Supporting Information (Figures S10–S12). These data allow for direct comparison of baseline and post-intervention anxiety-like behaviors across all experimental groups (Table S16).

At the molecular level, CPTSD induced significant upregulation of SERT, 5-HT3A, and MAO-A mRNA expression in the hippocampus. These molecular changes were accompanied by an increase in hippocampal 5-HT levels and a simultaneous decrease in DOPAC concentrations, indicating altered monoamine metabolism. In earlier studies where analyses were performed two weeks after PS exposure, PTSD was characterized by decreased hippocampal 5-HT [49,58,59]. In contrast, at the later time point analyzed here (three weeks post-stress), 5-HT levels were elevated, while anxiety remained high in both cases. These findings suggest that distinct molecular mechanisms sustain anxiety-like behavior at different stages of PTSD progression. During the early phase, anxiety-like behavior was associated with reduced DA and 5-HT levels coupled with increased NE concentrations in the hippocampus. At the later stage, however, anxiety correlated with elevated hippocampal 5-HT accompanied by increased 5-HT3AR, SERT, and MAO-A gene expression.

Recent studies employing an animal model of stress-related anxiety based on fox-urine cues have reported similar results, showing increased hippocampal 5-HT accompanied by elevated 5-HT3AR, SERT, and MAO-A expression [60]. In both cases, enhanced 5-HT3A gene expression may contribute to the development of anxiety-like behavior [61]. The 5-HT_3_ receptor, a ligand-gated ion channel expressed predominantly on interneurons throughout the brain [62,63], exhibits high densities in the amygdala and hippocampus across most species [64]. In our study, we additionally demonstrated increased 5-HT3A expression in the striatum of CPTSD rats.

Increased hippocampal 5-HT levels were accompanied by elevated SERT gene expression. SERT regulates synaptic 5-HT concentrations by reuptaking this monoamine into the presynaptic neuron, where it is subsequently metabolized by MAO-A [65,66]. Notably, in CPTSD rats, hippocampal MAO-A mRNA levels were also increased. Despite this upregulation, CPTSD induced a down-regulation of hippocampal DOPAC, a MAO-A-dependent DA metabolite. Hippocampal DOPAC may therefore serve as a potential biomarker of CPTSD resilience. This hypothesis is supported by its negative correlation with time spent in the dark arms (r=−0.9, p<0.05) and positive correlation with time spent in the open arms (r=−0.87, p<0.05) in CPTSD rats. Conversely, in untreated CPTSD animals, hippocampal MAO-A mRNA levels showed a strong negative correlation with time spent in the open arms (r=−0.97, p<0.05). In contrast, striatal MAO-A mRNA expression was negatively correlated with time spent in the closed arms (r=−0.88, p<0.05), indicating the involvement of MAO-A in the down-regulation of anxiety-like behavior in CPTSD. Expression of the D2R gene in the striatum may also contribute to attenuation of CPTSD-like anxiety, as evidenced by a negative correlation between D2R mRNA levels and time spent in closed arms (r=−0.9, p<0.05). Furthermore, striatal 5-HT3AR expression appeared to be associated with anxiety-like behavior, supported by its negative correlation with time spent in the open arms (r=−0.94, p<0.05). Finally, 5-HT3A gene expression in the hippocampus may be linked to depressive-like behavior, as indicated by its positive correlation with immobility time in the Porsolt swim test (r=0.95, p<0.05).

In CPTSD rats, depressive behavior also shows significant correlations with DA and 5-HT metabolites. Negative correlations were observed between immobility time in the Porsolt swimming test and both HVA (r=−0.9, p<0.05) and 5HIAA (r=−0.9, p<0.05) concentrations in the striatum. These findings indicate that, in untreated CPTSD rats, DA and 5-HT metabolites, as well as 5-HT3AR gene expression in the hippocampus, are associated with anxiety-related behavior, whereas in the striatum they are primarily linked with depressive-like behavior.

The central involvement of the striatum in PTSD is corroborated by its functional connectivity with limbic circuits, especially the amygdala and hippocampus [67]. In PTSD, traumatic memory processing preferentially activates the striatum while suppressing hippocampal engagement, effectively shifting behavioral control from hippocampal- to striatal-dependent memory systems. Consistent with this, fMRI investigations have demonstrated persistent hippocampal–striatal hyperconnectivity in PTSD, characterized by reduced flexibility and impaired reciprocal regulation [68]. Furthermore, lower striatal reactivity has been linked to heightened stress vulnerability in individuals subjected to early-life adversity [64]. Reciprocal pathways between the hippocampus and the dopaminergic striatal nucleus accumbens further emphasize these interregional dynamics, with widespread striatal expression of D1 and D2 receptors making these nuclei critical stress targets [69]. Here, CPTSD produced robust increases in D2R mRNA expression in the striatum. Behavioral correlations underscored their relevance: striatal D2R mRNA levels correlated positively with immobility duration in the Porsolt swim test (r=0.87, p<0.05) and negatively with BDNF expression (r=−0.85, p<0.05), implicating D2R as both a behavioral and molecular integrator of stress adaptation in CPTSD.

Analysis of neurochemical profiles within the striatum revealed marked perturbations in the principal neurotransmitter systems following stress and pharmacological intervention. The noradrenergic pathway exhibited the most substantial alteration, with norepinephrine concentrations reduced by 43% in the stress plus drug group relative to controls (2.50 vs. 4.35 ng/mg tissue, p<0.01). This pronounced decline likely reflects depletion of noradrenergic reserves consequent to prolonged stress exposure.

Significant changes were also evident in dopaminergic metabolism; levels of HVA, a major dopamine metabolite, were consistently diminished across all experimental cohorts, with the largest reduction observed in the PS+LK00764 group (47.4 vs. 94.8 ng/mg tissue in controls), indicating disrupted dopamine catabolism under these conditions. Dopamine itself and its primary metabolite DOPAC both trended downward in concentration, though these differences did not reach statistical significance.

In contrast, markers of the serotonergic system in the striatum remained relatively stable, with only minor intergroup fluctuations in 5-HT and 5HIAA levels. This suggests a predominant impact of stress and TAAR1 agonist administration on catecholaminergic, rather than serotonergic, neurotransmission within the striatal compartment.

Treatment with LK00764 resulted in a complete reversal of CPTSD-induced behavioral abnormalities, as evidenced by reductions in passive immobility during the Porsolt swim test and normalization of anxiety-related behaviors in the EPM. Specifically, this included decreased time spent in the closed arms and increased exploration of the open arms. While LK00764 alone elicited modest anxiolytic effects, its impact under stress conditions was more pronounced, especially in normalizing SERT and BDNF expression. This synergistic effect is reminiscent of clinical observations with conventional antidepressants, which often demonstrate enhanced therapeutic efficacy in subjects sensitized by chronic stress.

Neurochemically, the hallmark effects of LK00764 treatment in CPTSD rats were reductions in hippocampal 5-HT and in NE and DA concentrations within the striatum. Notably, significant decreases in DOPAC and HVA were observed only in the striatum after treatment, while these changes were absent in the hippocampus. Unstressed animals treated with LK00764 mirrored these neurochemical patterns, reinforcing the notion that TAAR1 influences both the dopaminergic and serotonergic systems, as recognized in various behavioral disorders.

A key insight is the proposed role of TAAR1-mediated modulation of dopamine metabolism, particularly through catecholamine-*O*-methyltransferase (COMT), a pivotal enzyme in dopamine and norepinephrine catabolism throughout the CNS and periphery [70]. In unstressed LK00764-treated rats, COMT mRNA levels correlated positively with striatal norepinephrine (r=0.73; p<0.05), dopamine (r=0.73; p<0.05), DOPAC (r=0.8; p<0.05), and HVA (r=0.71; p<0.05) concentrations. Intriguingly, LK00764 downregulated COMT gene expression in unstressed animals but upregulated it under CPTSD conditions. Furthermore, in treated CPTSD rats, an inverse association was found between COMT mRNA and time spent in closed arms in the EPM (r=0.8; p<0.05), supporting COMT as a potential mechanistic target underlying the behavioral benefits of TAAR1 agonism.

Additional gene targets included D2R, whose expression in the striatum was diminished following LK00764 therapy. A positive correlation emerged between D2R mRNA and behavioral despair (immobility) in the Porsolt swim test for treated CPTSD animals (r=0.82; p<0.05), indicating a possible link between reduced D2R expression and therapeutic outcomes. Importantly, LK00764 robustly upregulated BDNF gene expression in the striatum, a neuroplasticity marker crucial for synaptic remodeling and resilience. This effect is further supported by the observed positive correlation (r=0.65; p<0.05) between striatal BDNF mRNA and time spent in open arms in the EPM among treated CPTSD rats. Indirect evidence also connects TAAR1 agonism with prevention of synaptic depression via the BDNF-TrκB pathway, providing a mechanistic rationale for its protective neurobehavioral effects.

For a comprehensive discussion of region-specific molecular and behavioral effects of LK00764, including cluster-level analyses of striatal and hippocampal biomarkers, readers are referred to the Supporting Information.

The principal novelty of our study lies in the identification of the TAAR1 agonist’s capacity to enhance MAO-A expression in the liver of CPTSD rats. Untreated CPTSD animals exhibited an upward trend in hepatic MAO-A mRNA levels. Notably, a significant negative correlation was observed between hepatic MAO-A mRNA expression and immobility time in the Porsolt test (r=−0.8; p<0.05). These findings suggest that LK00764 potentiates post-stressor changes in the liver aimed at mitigating stress-related behaviors.

Importantly, hepatic MAO-A catalyzes the oxidative deamination of gut-derived monoamines produced via microbial amino acid decarboxylation [71,72]. Therefore, it can be posited that hepatic MAO-A plays a pivotal role in regulating the gut-liver-brain axis by modulating levels of trace amines such as tyramine, tryptamine, phenylethylamine, and others. These amines enter the liver through the portal vein, where they are partially metabolized by hepatic MAO-A prior to entering the systemic circulation and subsequently crossing the blood-brain barrier.

The clinical significance of our findings resides in the potential application of identified biomarker profiles for diagnosing stress-related disorders and monitoring therapeutic efficacy. The region-specific neurochemical alterations observed underscore the necessity for tailored interventions addressing distinct components of the stress response.

A comprehensive understanding of the molecular mechanisms underlying the protective effects of TAAR1 agonists in CPTSD necessitates detailed reconstruction of signaling cascades from receptor engagement to downstream targets such as BDNF. In this study, we delineated a discrete gene network hub centered on TAAR1, encompassing coexpressed genes, their protein products, and gene–protein interactions forming a closed regulatory loop. This network incorporates genes profiled herein, alongside novel candidates yet to be explored in the context of CPTSD.

Notably, the reconstructed hub includes PARK2 (parkin), ERBB4, and OPRM1. PARK2 encodes parkin, a pivotal regulator of mitochondrial quality control and dopaminergic neuron function, with genetic variants implicated in heightened PTSD risk mediated via dysfunction in dopamine signaling and stress pathway regulation [73]. ERBB4 modulates fear and anxiety through regulation of GABAergic interneurons within the amygdala and prefrontal cortex, with aberrations linked to PTSD-like phenotypes [73]. Although ERBB4 association with PTSD remains underexplored, genetic linkage between TAAR1 and OPRM1 is established [74,75,76]. OPRM1 encodes the μ-opioid receptor, where the A118G polymorphism modulates individual stress responsiveness and PTSD susceptibility via opioid neurotransmission, emotional pain processing, and hypothalamic–pituitary–adrenal axis regulation [74]. Future investigations are required to experimentally validate the involvement of these genes within PTSD pathogenesis and to assess their integrative role in the TAAR1-centered gene network.

Collectively, the data affirm the systemic nature of stress-induced alterations spanning molecular, neurochemical, and behavioral strata. The serotonergic system and monoamine metabolism genes, prominently MAO-A, displayed the highest molecular sensitivity. Neurochemical perturbations were most notable within noradrenergic and dopaminergic circuits in the striatum. Behaviorally, animals exhibited depressive- and anxiety-like phenotypes, with the striatum demonstrating superior molecular responsiveness relative to the hippocampus.

### Limitations

Several limitations of the present study should be acknowledged. First, although effect sizes were systematically quantified, the study was conducted using relatively small and unequal group sizes, which may limit statistical power for certain comparisons. Second, only male rats were included, preventing assessment of potential sex-specific differences in behavioral or molecular responses—an important consideration given documented sex differences in stress reactivity and CPTSD vulnerability.

Third, the molecular characterization was restricted to mRNA expression levels without complementary protein-level validation. While transcriptional changes offer valuable mechanistic insight, post-transcriptional regulation and protein turnover may produce divergent functional outcomes, underscoring the need for future proteomic or immunohistochemical analyses.

Fourth, the TAAR1-centered gene network reconstruction presented here remains hypothetical and requires experimental verification through targeted perturbation studies or cell-type–specific assays. Finally, the current investigation did not include the amygdala, a key regulator of fear and anxiety circuits. Its exclusion limits the anatomical scope of the findings and leaves open questions regarding region-specific TAAR1-dependent mechanisms within canonical stress–anxiety networks.

Despite these limitations, the multi-level integration of behavioral, neurochemical, and transcriptional data provides a coherent framework for understanding TAAR1-mediated modulation of stress-related phenotypes and establishes a foundation for more comprehensive future investigations.

## 5. Conclusions

This study provides pioneering evidence for the efficacy of the TAAR1 agonist LK00764 in a rodent model of CPTSD. Post-traumatic administration of LK00764 robustly attenuated anxiety-like and depression-like behaviors in CPTSD, supporting its therapeutic potential beyond simple PTSD. Mechanistically, the protective effects were associated with upregulation of striatal BDNF and COMT gene expression and marked reduction in hippocampal 5-HT content, suggesting region-specific molecular adaptations.

Our analyses identified novel molecular targets in the hippocampus and striatum, particularly highlighting the centrality of gene network disruptions in PTSD pathogenesis. The serotonergic system emerged as most responsive to stress and drug intervention, with the strongest modulation observed in SERT and 5-HT3AR gene expression and hippocampal 5-HT levels. These results advocate for the utility of gene network analysis in delineating critical pathways for pharmacological targeting in PTSD.

A reconstructed TAAR1-centered gene network hub revealed new candidate genes, including parkin, not previously implicated in PTSD, identified through artificial intelligence-aided approaches. Future experiments are warranted to validate their roles and refine our understanding of TAAR1-dependent signaling in stress-related pathology.

Clinically, these results underscore the translational relevance of molecular markers such as MAO-A, SERT, and 5-HT3AR as potential therapeutic targets. While LK00764 displayed advantageous behavioral effects, further research on the durability and long-term impact of enhanced molecular changes remains essential. Overall, the findings highlight the importance of a multilevel integrative approach, linking molecular, neurochemical, and behavioral domains to unravel the complex neurobiology of stress responses and inform rational development of novel therapeutics.

## Figures and Tables

**Table 1:** The primer sequences used for the evaluation of mRNA expression levels.

Primer Name	Primer Sequence (5’ ⟶ 3’)	Temperature (°C)
18S	**F** ACGGACCAGAGCGAAAGCAT **R** TGTCAATCCTGTCCGTGTCC	60
GAPDH	**F** AAACCCATCACCATCTTCCA **R** GTGGTTCACACCCATCACAA	60
5-HT3	**F** CTGTCCTCCATCCGCCACTCC **R** CAGCAGCCTGTCCAGCACATATC	60
SERT	**F** ATAGCCAACATGCCAGCATCCAC **R** ACCACGATGAGCACGAACCATTC	68
MAO-A	**F** GCCAGGAACGGAAATTTGTA **R** TCTCAGGTGGAAGCTCTGGT	64
MAO-B	**F** TGGGAAGATTCCAGAGGATG **R** GCTGACAAGATGGTGGTCAA	60
BDNF	**F** GAAAGTCCCGGTATCAAAAG **R** CGCCAGCCAATTCTCTTTTTG	60
COMT-105	**F** CTGGAGAAATGTGGCCTGCT **R** GCTGCTGCTCCCTCTCACAT	60

**Table 2:** CONSORT-style summary of group sizes, exclusions, and final sample numbers used in behavioral tests (EPM/PST) and biochemical analysis (PCR and HPLC).

Group	Initial *n*	Excluded	EPM/PST/PCR *n*	Excluded	HPLC (Striatum/ Hippocampus) *n*
control	10	1 *	9	1/0 **	8/9
LK00764	10	2 *	8	0/2 **	8/6
PS	10	4 *	6	1/1 **	5/5
PS+LK00764	10	3 *	7	0/0	7/7

* = high-anxiety phenotype at baseline; ** = technical failure.

**Table 3:** Behavioral parameters measured in the Porsolt swim test and the elevated plus maze across experimental groups. Data are presented as mean ± SEM.

Parameters	Control (*n* = 9)	LK00764 (*n* = 8)	PS (*n* = 6)	PS+LK00764 (*n* = 7)
Active swim	43.19 ± 5.79	54.62 ± 6.41 *^,###^	27.89 ± 10.57 **	41.36 ± 8.96 *
Passive swim	45.38 ± 10.15	36.31 ± 11.29 ^##^	59.37 ± 7.62	44.88 ± 10.37
Dives	1.30 ± 1.16	1.29 ± 1.05	1.28 ± 1.21	2.31 ± 3.05
Climbs	10.13 ± 6.30	7.78 ± 5.38	11.46 ± 6.70	11.46 ± 8.79
Open arms	41.79 ± 16.65	64.37 ± 23.19 ^###^	7.86 ± 14.44 *	26.26 ± 14.20
Closed arms	38.52 ± 5.2	27.91 ± 8.9 ^#^	65.65 ± 9.34 **	53.96 ± 8.24 *
Center	19.67 ± 7.8	7.71 ± 0.83	26.49 ± 7.28	19.67 ± 6.29

* = effect compared to control group; ^#^ = effect compared to PS group; * *p* < 0.05; ** *p* < 0.01; ^#^ *p* < 0.05; ^##^ *p* < 0.01; ^###^ *p* < 0.001.

## Data Availability

The original contributions presented in the study are included in the article/Supplementary Materials, further inquiries can be directed to the corresponding authors.

## Supplementary Materials

The following supporting information can be downloaded at: https://www.mdpi.com/article/10.3390/biomedicines13122972/s1.
