# Supplementary material for: New Insights into Complex PTSD Treatment: Focus on TAAR1 Agonists
Source: Biomedicines. 2025 Dec 3;13(12):2972. doi: 10.3390/biomedicines13122972 (PMC12730721; doi:10.3390/biomedicines13122972)
Supplement: Supplementary file 1 [file biomedicines-13-02972-s001.zip › biomedicines-3952830-supplementary.pdf]

Supplementary Materials: New Insights into Complex PTSD Treatment: Focus on TAAR1 Agonists

1. Discussion

Overall, LK00764 exhibited dual effects. At the behavioral level, the compound displayed significant antidepressant and anxiolytic actions, with treatment improving outcomes across both anxiety- and depression-related paradigms. Intriguingly, at the molecular level, the combination of stress exposure and LK00764 frequently enhanced the magnitude of gene expression alterations, likely reflecting an activation of compensatory and adaptive transcriptional mechanisms.

Cluster-level analysis of median biomarker values highlighted region-specific molecular signatures of stress. In the striatum, the most informative indicators were BDNF, MAO-A, and COMT, with stress-related clusters showing a two- to three-fold elevation relative to controls–signifying robust neurotrophic and catecholaminergic response. Within the hippocampus, key biomarkers included BDNF, the 5-HT transporter SERT, and dopamine transporter DAT, with coordinated upregulation of the serotonergic pathway supported by increased SERT expression and heightened levels of the metabolite 5HIAA.

Drug treatment exerted regionally selective effects; in the striatum, pharmacological intervention partially normalized stress-induced molecular changes, resulting in the emergence of a discrete "stress + drug" cluster characterized by intermediate biomarker values. Conversely, in the hippocampus, LK00764 administration did not produce a distinct clustering effect, underscoring limited impact on stress-driven neurochemical adaptations within this region.

Integrated analysis underscored the preeminent influence of stress as the principal determinant of neuronal alterations across both examined brain regions. The distinct clustering of animals based on stress exposure status highlights the systemic and coordinated nature of neurobiological adaptations to chronic stress.

The observed clustering patterns embody fundamental organizational principles of the mammalian stress response. The striatum, a key regulator of motor function and motivational drives, exhibited notable plasticity and heterogeneity in its reaction profiles, reflecting its pivotal role in shaping adaptive behavioral responses under varying environmental challenges.

2. Tables

Table S1. One-way ANOVA summary for behavioral parameters in the Porsolt forced swim test. Statistically significant p-values (<0.05) are shown in bold.

Variable	$F_{3,26}$	p	η^2
Dives	0.59	0.628	0.064
Climbs	0.48	0.698	0.053
Active swim	13.40	**< 0.001**	0.607
Passive swim	5.99	**0.003**	0.409

Table S2. Tukey test summary for behavioral parameters in the Porsolt forced swim test. Statistically significant adjusted *p*-values (<0.05) are shown in bold.

Variable	Test	Group 1	Group 2	p_{adj}
dives	Tukey HSD	LK00764	PS	1
		LK00764	PS+LK00764	0.685
		LK00764	control	1
		PS	PS+LK00764	0.725
		PS	control	1
		PS+LK00764	control	0.678
climbs	Tukey HSD	LK00764	PS	0.751
		LK00764	PS+LK00764	0.727
		LK00764	control	0.892
		PS	PS+LK00764	1
		PS	control	0.982
		PS+LK00764	control	0.980
active_swim	Tukey HSD	LK00764	PS	**< 0.001**
		LK00764	PS+LK00764	**0.015**
		LK00764	control	**0.028**
		PS	PS+LK00764	**0.023**
		PS	control	**0.005**
		PS+LK00764	control	0.966
passive_swim	Tukey HSD	LK00764	PS	**0.001**
		LK00764	PS+LK00764	0.375
		LK00764	control	0.275
		PS	PS+LK00764	0.071
		PS	control	0.064
		PS+LK00764	control	0.100

Table S3. Results of two-way ANOVA showing main effects and interaction of LK00764 and PS on the measured Porsolt forced swim test variables. Values in the *p* column less than 0.05 are highlighted in bold.

Dependent	Test	Effect	$F_{(1,26)}$	p	η^2
dives	ANOVA	LK00764	0.454	0.506	0.016
		PS	0.600	0.446	0.022
		LK00764*PS	0.638	0.432	0.023
climbs	ANOVA	LK00764	0.287	0.597	0.010
		PS	1.005	0.325	0.037
		LK00764*PS	0.218	0.644	0.008
active_swim	ANOVA	LK00764	18.448	**<0.001**	0.268
		PS	24.307	**<0.001**	0.353
		LK00764*PS	0.123	0.728	0.002
passive_swim	ANOVA	LK00764	9.539	**0.005**	0.211
		PS	9.071	**0.006**	0.201
		LK00764*PS	0.530	0.473	0.012

Table S4. Summary of Kruskal–Wallis test results for behavioral parameters measured in the elevated plus maze. Statistically significant p-values (<0.05) are highlighted in bold.

Variable	H	p	η^2_{KW}
closed	15.653	**0.001**	0.540
open	19.980	**<0.001**	0.689
center	7.096	0.069	0.245

Table S5. Results of pairwise Dunn post hoc tests for behavioral outcomes in the elevated plus maze, with adjusted p-values indicating significant differences between experimental groups.

Variable	Test	Group 1	Group 2	p_{adj}
closed	Dunn	PS	LK00764	**0.038**
		PS+LK00764	LK00764	0.104
		control	LK00764	1
		PS+LK00764	PS	1
		control	PS	**0.010**
		control	PS+LK00764	**0.029**
open	Dunn	PS	LK00764	**<0.001**
		PS+LK00764	LK00764	**0.010**
		control	LK00764	0.940
		PS+LK00764	PS	1
		control	PS	**0.028**
		control	PS+LK00764	0.367
center	Dunn	PS	LK00764	0.100
		PS+LK00764	LK00764	0.297
		control	LK00764	0.301
		PS+LK00764	PS	1
		control	PS	1
		control	PS+LK00764	1

Table S6. ANOVA results for striatal concentrations of neurotransmitters and metabolites, showing F-statistics (F), p-values (p), and η^2 effect sizes. Statistically significant p-values (<0.05) are highlighted in bold.

Compound	Test	$F_{(3,24)}$	p	η^2
NE	ANOVA	4.027	**0.019**	0.335
DOPAC	ANOVA	2.207	0.113	0.216
5HIAA	ANOVA	1.428	0.259	0.151
DA	ANOVA	3.071	**0.047**	0.277
HVA	ANOVA	3.579	**0.029**	0.309
5-HT	ANOVA	1.472	0.247	0.155

Table S7. Pairwise Tukey post hoc comparisons of neurotransmitter and metabolite concentrations in the striatum, with adjusted p-values indicating significant differences between experimental groups.

Compound	Test	Group 1	Group 2	p_{adj}
NE	Tukey HSD	LK00764	PS	0.571
		LK00764	PS+LK00764	0.555
		LK00764	control	0.229
		PS	PS+LK00764	0.100
		PS	control	0.971
		PS+LK00764	control	**0.018**
DOPAC	Tukey HSD	LK00764	PS	1.000
		LK00764	PS+LK00764	0.464
		LK00764	control	0.676
		PS	PS+LK00764	0.584
		PS	control	0.741
		PS+LK00764	control	0.074
5HIAA	Tukey HSD	LK00764	PS	0.825
		LK00764	PS+LK00764	0.851
		LK00764	control	0.694
		PS	PS+LK00764	0.425
		PS	control	1.000
		PS+LK00764	control	0.269
DA	Tukey HSD	LK00764	PS	0.991
		LK00764	PS+LK00764	0.998
		LK00764	control	0.081
		PS	PS+LK00764	0.969
		PS	control	0.246
		PS+LK00764	control	0.066
HVA	Tukey HSD	LK00764	PS	0.930
		LK00764	PS+LK00764	0.960
		LK00764	control	0.070
		PS	PS+LK00764	0.738
		PS	control	0.358
		PS+LK00764	control	**0.029**
5-HT	Tukey HSD	LK00764	PS	0.825
		LK00764	PS+LK00764	0.986
		LK00764	control	0.406
		PS	PS+LK00764	0.668
		PS	control	0.951
		PS+LK00764	control	0.264

Table S8. Two-way ANOVA analysis of neurotransmitter and metabolite concentration changes in the striatum. Statistically significant *p*-values (<0.05) are highlighted in bold.

Dependent	Test	Effect	$F_{(1,25)}$	p	η^2
NE	ANOVA	LK00764	9.394	**0.005**	0.266
		PS	1.638	0.213	0.046
		LK00764*PS	0.319	0.577	0.009
DOPAC	ANOVA	LK00764	2.852	0.104	0.095
		PS	3.170	0.088	0.105
		LK00764*PS	0.058	0.812	0.002
5HIAA	ANOVA	LK00764	3.393	0.078	0.121
		PS	0.442	0.512	0.016
		LK00764*PS	0.217	0.646	0.008
DA	ANOVA	LK00764	4.910	**0.036**	0.150
		PS	2.044	0.166	0.063
		LK00764*PS	1.683	0.207	0.052
HVA	ANOVA	LK00764	6.970	**0.014**	0.205
		PS	2.219	0.149	0.065
		LK00764*PS	0.829	0.372	0.024
5-HT	ANOVA	LK00764	3.790	0.063	0.134
		PS	0.375	0.546	0.013
		LK00764*PS	0.026	0.873	0.001

Table S9. One-way ANOVA and Kruskal–Wallis (KW) test results for hippocampal neurotransmitter and metabolite concentrations. Statistically significant *p*-values (<0.05) are highlighted in bold.

Compound	Test	$F_{(3,23)}$ / H	p	η^2 / η^2_{KW}
NE	ANOVA	2.297	0.104	0.231
DOPAC	ANOVA	1.641	0.207	0.176
5HIAA	ANOVA	8.309	**0.040**	0.320
DA	KW	2.898	0.408	0.111
HVA	ANOVA	1.087	0.375	0.124
5-HT	ANOVA	7.533	**<0.001**	0.496

Table S10. Tukey HSD and Dunn post hoc comparisons of hippocampal neurotransmitters and metabolites. Bold indicates $p_{adj} < 0.05$.

Compound	Test	Group 1	Group 2	p_{adj}
NE	Tukey HSD	LK00764	PS	0.767
		LK00764	PS+LK00764	0.642
		LK00764	control	0.756
		PS	PS+LK00764	0.173
		PS	control	0.999
		PS+LK00764	control	0.117
DOPAC	Tukey HSD	LK00764	PS	0.889
		LK00764	PS+LK00764	1.000
		LK00764	control	0.524
		PS	PS+LK00764	0.852
		PS	control	0.188
		PS+LK00764	control	0.529
5HIAA	Dunn	LK00764	PS	0.883
		LK00764	PS+LK00764	0.628
		LK00764	control	**0.024**
		PS	PS+LK00764	1.000
		PS	control	1.000
		PS+LK00764	control	1.000
DA	Dunn	LK00764	PS	1.000
		LK00764	PS+LK00764	1.000
		LK00764	control	0.821
		PS	PS+LK00764	1.000
		PS	control	1.000
		PS+LK00764	control	1.000
HVA	Tukey HSD	LK00764	PS	0.474
		LK00764	PS+LK00764	0.376
		LK00764	control	0.672
		PS	PS+LK00764	1.000
		PS	control	0.954
		PS+LK00764	control	0.923
5-HT	Tukey HSD	LK00764	PS	**<0.001**
		LK00764	PS+LK00764	0.245
		LK00764	control	**0.037**
		PS	PS+LK00764	**0.031**
		PS	control	0.124
		PS+LK00764	control	0.796

Table S11. One-way ANOVA and Kruskal–Wallis (KW) test analysis of striatal mRNA expression. Statistically significant p-values (<0.05) are highlighted in bold, and effect sizes are reported as η^2 and η^2_{KW}.

Gene	Test	$F_{(3,26)}$ / H	p	η^2 / η^2_{KW}
BDNF	KW	16.056	**0.001**	0.554
D2R	KW	9.043	**0.029**	0.312
MAO-A	KW	21.420	**<0.001**	0.739
MAO-B	KW	0.593	0.898	0.020
COMT	KW	16.653	**<0.001**	0.574
5-HT3AR	ANOVA	24.879	**<0.001**	0.742
SERT	KW	20.657	**<0.001**	0.712

Table S12. Pairwise Dunn post hoc test results for striatal mRNA expression of neurotransmission-related genes. Adjusted p-values less than 0.05, indicating statistically significant differences between groups, are highlighted in bold.

Gene	Test	Group 1	Group 2	p_{adj}
BDNF	Dunn	LK00764	PS	1.000
		LK00764	PS+LK00764	**0.048**
		LK00764	control	1.000
		PS	PS+LK00764	**0.001**
		PS	control	1.000
		PS+LK00764	control	**0.009**
D2R	Dunn	LK00764	PS	**0.037**
		LK00764	PS+LK00764	1.000
		LK00764	control	1.000
		PS	PS+LK00764	0.179
		PS	control	0.091
		PS+LK00764	control	1.000
MAO-A	Dunn	LK00764	PS	**0.015**
		LK00764	PS+LK00764	**0.006**
		LK00764	control	1.000
		PS	PS+LK00764	1.000
		PS	control	**0.008**
		PS+LK00764	control	**0.003**
MAO-B	Dunn	LK00764	PS	1.000
		LK00764	PS+LK00764	1.000
		LK00764	control	1.000
		PS	PS+LK00764	1.000
		PS	control	1.000
		PS+LK00764	control	1.000
COMT	Dunn	LK00764	PS	**0.023**
		LK00764	PS+LK00764	**<0.001**
		LK00764	control	0.353
		PS	PS+LK00764	1.000
		PS	control	1.000
		PS+LK00764	control	0.207
5-HT3AR	Tukey HSD	LK00764	PS	**0.001**
		LK00764	PS+LK00764	**<0.001**
		LK00764	control	1.000
		PS	PS+LK00764	0.109
		PS	control	**<0.001**
		PS+LK00764	control	**<0.001**
SERT	Dunn	LK00764	PS	0.156
		LK00764	PS+LK00764	**0.007**
		LK00764	control	1.000
		PS	PS+LK00764	1.000
		PS	control	**0.024**
		PS+LK00764	control	**<0.001**

Table S13. Kruskal–Wallis (KW) and one-way ANOVA analysis of hippocampal neurotransmission-related gene expression. Statistically significant p-values (<0.05) are highlighted in bold, and effect sizes are reported as η^2_{KW} for KW tests and η^2 for ANOVA.

Gene	Test	H / $F_{(3,26)}$	p_{adj} / p	η^2_{KW} / η^2
BDNF	ANOVA	1.661	0.200	0.161
SERT	KW	8.696	**0.034**	0.300
COMT	KW	8.027	0.045	0.277
5-HT3A	KW	8.588	**0.035**	0.296
DAT	KW	< 0.001	1.000	< 0.001
MAO-A	ANOVA	13.281	**<0.001**	0.606
MAO-B	ANOVA	0.081	0.970	0.009

Table S14. Pairwise Dunn and Tukey HSD post hoc comparisons of neurotransmission-related gene expression in the hippocampus. Statistically significant adjusted p-values (<0.05) are highlighted in bold.

Gene	Test	Group 1	Group 2	p_{adj}
BDNF	Tukey HSD	LK00764	PS	0.727
		LK00764	PS+LK00764	0.803
		LK00764	control	0.796
		PS	PS+LK00764	0.998
		PS	control	0.247
		PS+LK00764	control	0.291
SERT	Dunn	LK00764	PS	0.068
		LK00764	PS+LK00764	1.000
		LK00764	control	1.000
		PS	PS+LK00764	0.333
		PS	control	**0.041**
		PS+LK00764	control	1.000
COMT	Dunn	LK00764	PS	0.688
		LK00764	PS+LK00764	1.000
		LK00764	control	1.000
		PS	PS+LK00764	0.410
		PS	control	**0.029**
		PS+LK00764	control	1.000
5-HT3AR		LK00764	PS	0.292
		LK00764	PS+LK00764	1.000
		LK00764	control	1.000
		PS	PS+LK00764	1.000
		PS	control	**0.025**
		PS+LK00764	control	0.759
DAT	Dunn	LK00764	PS	1.000
		LK00764	PS+LK00764	1.000
		LK00764	control	1.000
		PS	PS+LK00764	1.000
		PS	control	1.000
		PS+LK00764	control	1.000
MAO-A	Tukey HSD	LK00764	PS	**< 0.001**
		LK00764	PS+LK00764	0.204
		LK00764	control	0.959
		PS	PS+LK00764	0.018
		PS	control	**< 0.001**
		PS+LK00764	control	0.074
MAO-B	Tukey HSD	LK00764	PS	0.999
		LK00764	PS+LK00764	0.991
		LK00764	control	0.965
		PS	PS+LK00764	0.999
		PS	control	0.990
		PS+LK00764	control	0.999

Table S15. Pairwise Dunn post hoc comparisons of MAO-A mRNA expression in the liver across experimental groups. Adjusted p-values (p_{adj}) are reported; values below 0.05 would be highlighted in bold.

Gene	Test	Group 1	Group 2	p_{adj}
MAO-A	Dunn	LK00764	PS	0.298
		LK00764	PS+LK00764	1.000
		LK00764	control	0.299
		PS	PS+LK00764	0.150
		PS	control	1.000
		PS+LK00764	control	0.142

Table S16. Results of paired comparisons (paired t-test or Wilcoxon signed-rank test) for EPM measures across groups. Significant p-values ($p < 0.05$) are shown in bold.

Group	EPM parameter	Test	p-value	Statistic
control	closed	t-test	0.210	-1.379
	open	Wilcoxon	0.844	16.000
	center	t-test	0.079	2.055
LK00764	closed	t-test	**0.025**	2.848
	open	t-test	**< 0.001**	-6.074
	center	t-test	**< 0.001**	5.883
PS	closed	t-test	0.082	2.168
	open	t-test	**0.022**	-3.298
	center	t-test	0.087	2.123
PS+LK00764	closed	t-test	0.133	1.736
	open	t-test	**0.016**	-3.333
	center	t-test	**0.014**	3.428

3. Figures

Disclaimer/Publisher's Note: The statements, opinions and data contained in all publications are solely those of the individual author(s) and contributor(s) and not of MDPI and/or the editor(s). MDPI and/or the editor(s) disclaim responsibility for any injury to people or property resulting from any ideas, methods, instructions or products referred to in the content.

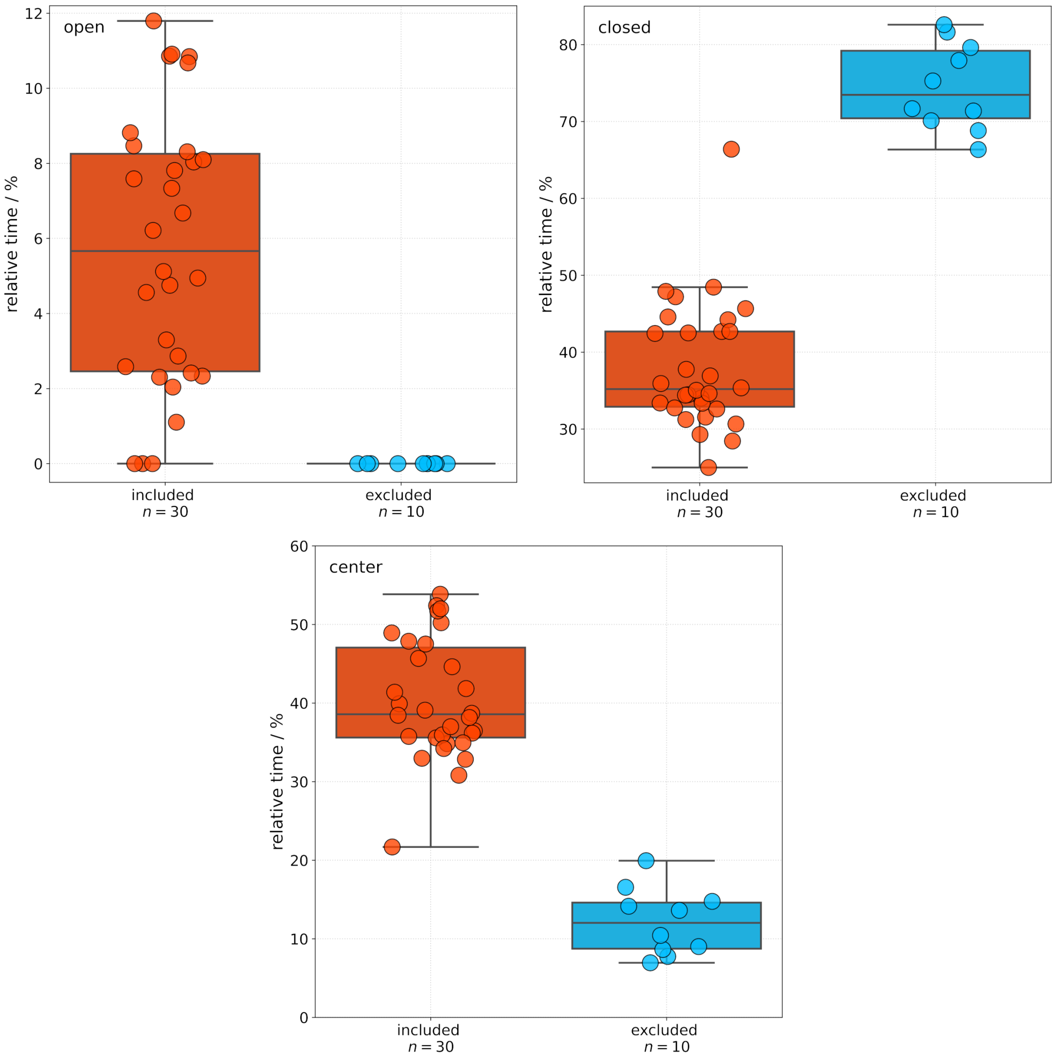

Figure S1. Boxplots of baseline elevated plus maze performance illustrating time spent in the open arms, closed arms, and center zone prior to stress exposure. These measurements were used to classify animals according to their initial anxiety phenotype and to determine their eligibility for further participation in the experiment (included vs. excluded).

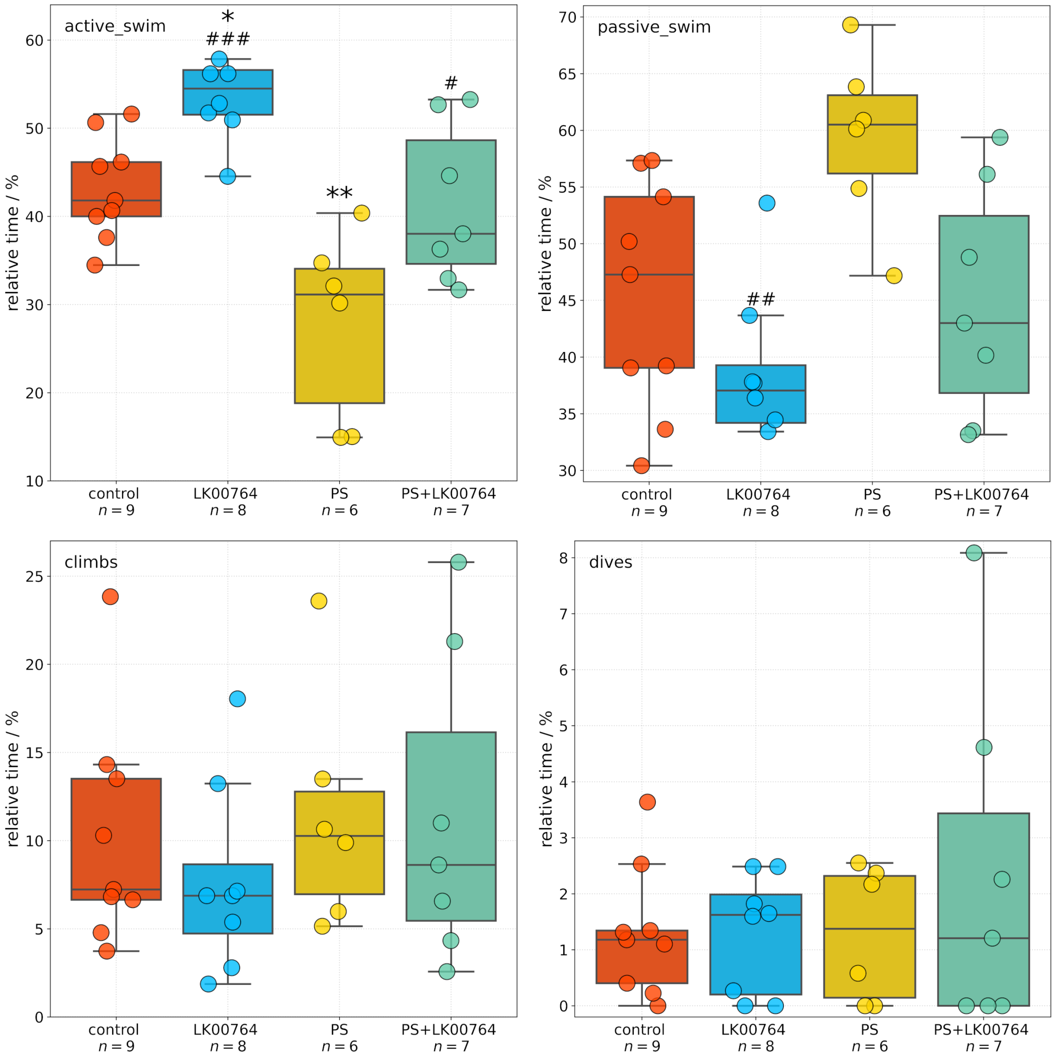

Figure S2. Boxplots of behavioral parameters in the Porsolt forced swim test showing group differences in diving, climbing, and swimming behaviors. Statistically significant differences between groups are indicated. Statistically significant differences between groups are indicated as * = effect compared to Control group; # = effect compared to PS group; * $p < 0.05$; ** $p < 0.01$; # $p < 0.05$; ## $p < 0.01$; ### $p < 0.001$.

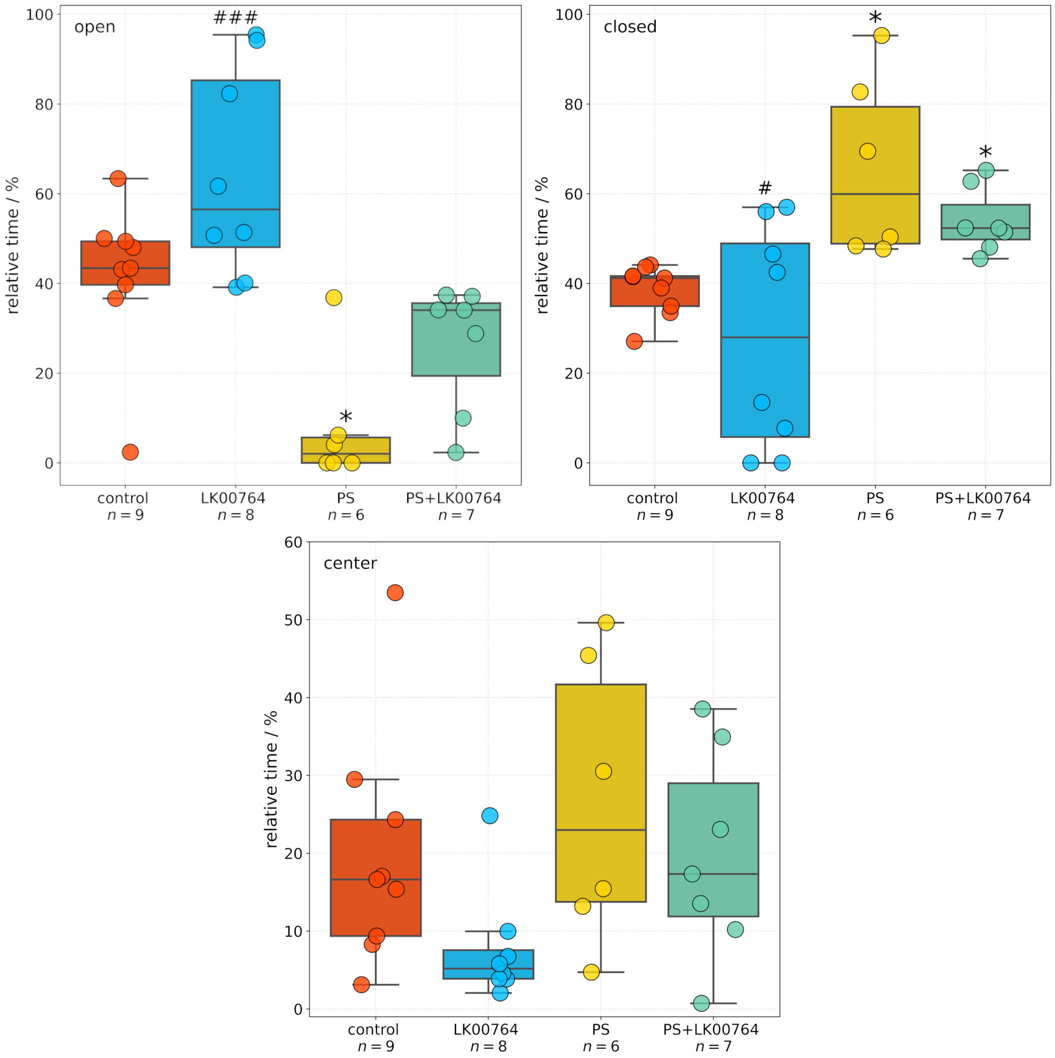

Figure S3. Boxplots of behavioral parameters in the elevated plus maze showing time spent in open arms, closed arms, and the center zone across treatment and stress groups. Statistically significant differences between groups are indicated as * = effect compared to Control group; # = effect compared to PS group; * $p < 0.05$; # $p < 0.05$; ### $p < 0.001$.

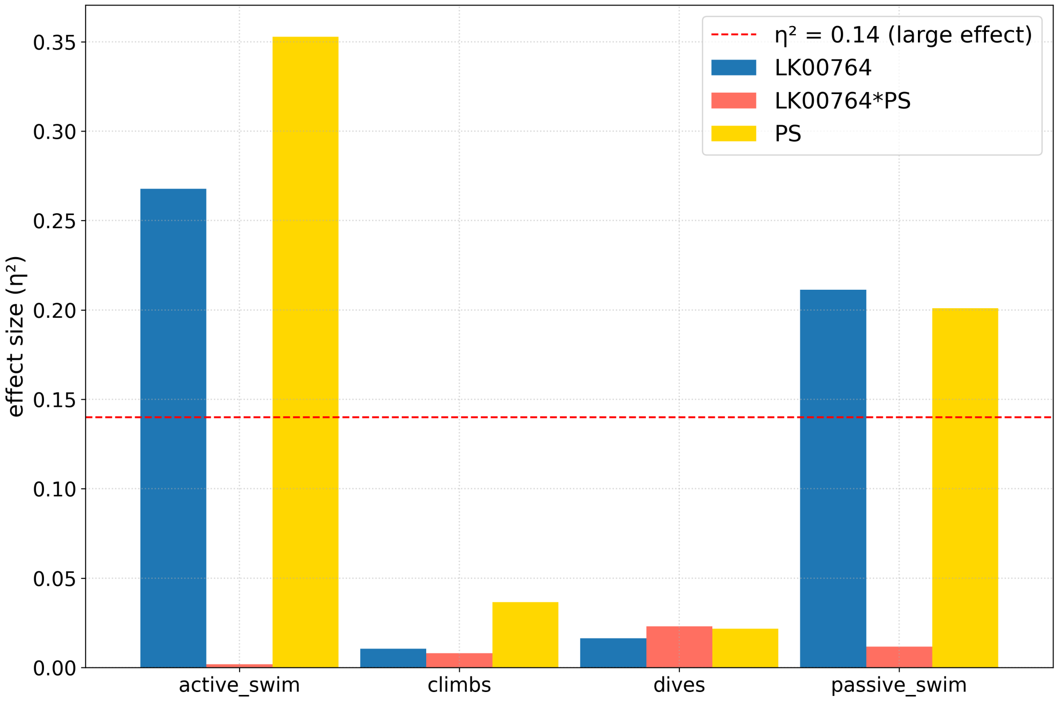

Figure S4. Effect size (η^2) estimated by two-way ANOVA for behavioral parameters in the Porsolt forced swim test. Bars represent the magnitude of main effects (predator stress and LK00764) and their interaction. The dashed red line indicates a large effect threshold ($\eta^2 = 0.14$).

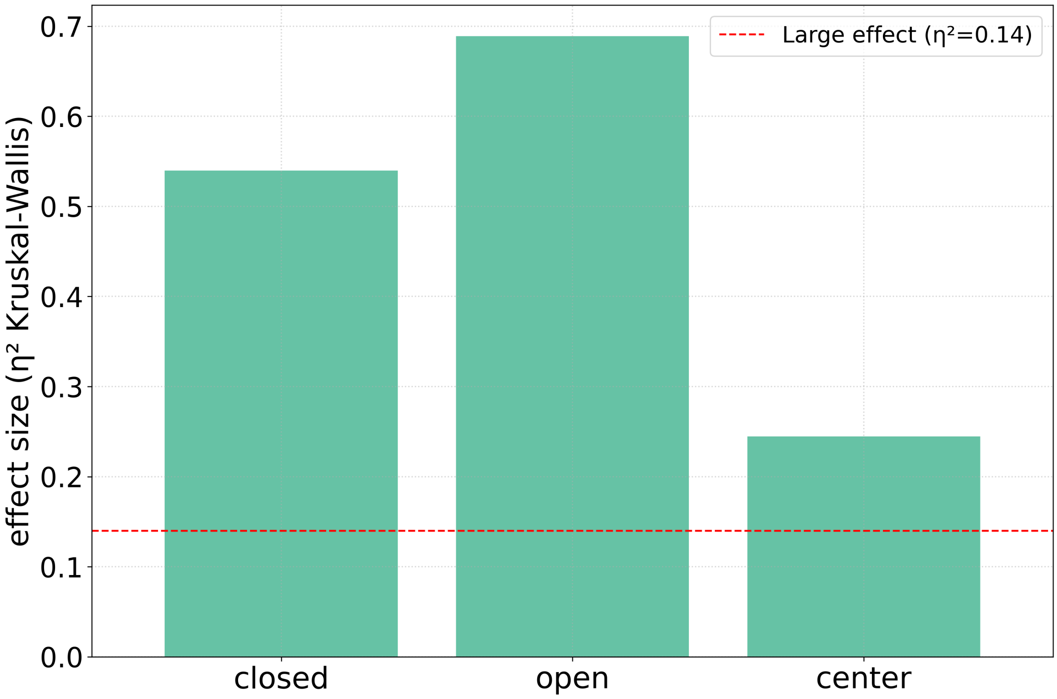

Figure S5. Effect sizes (η^2) derived from the Kruskal–Wallis test for behavioral measures obtained in the elevated plus maze. Higher η^2 values indicate stronger group-related differences across behavioral parameters. The dashed red line indicates a large effect threshold ($\eta^2 = 0.14$).

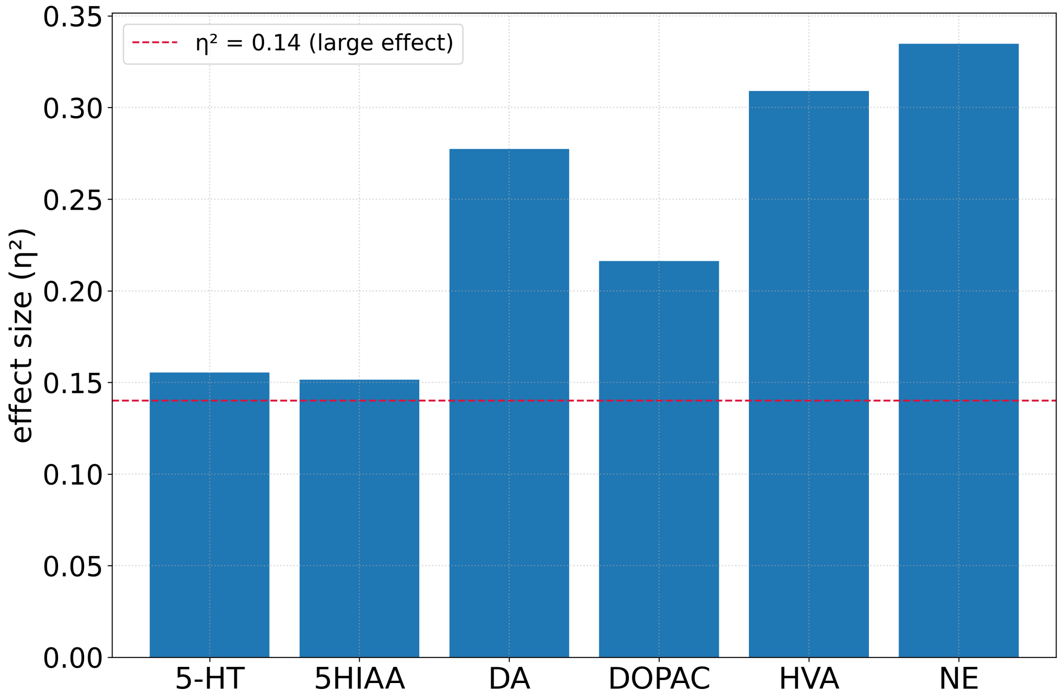

Figure S6. Effect size (η^2) computed from one-way ANOVA for neurotransmitter and metabolite concentrations in the striatum. Bars illustrate the magnitude of treatment effects, with the dashed red line denoting a large effect threshold ($\eta^2 = 0.14$).

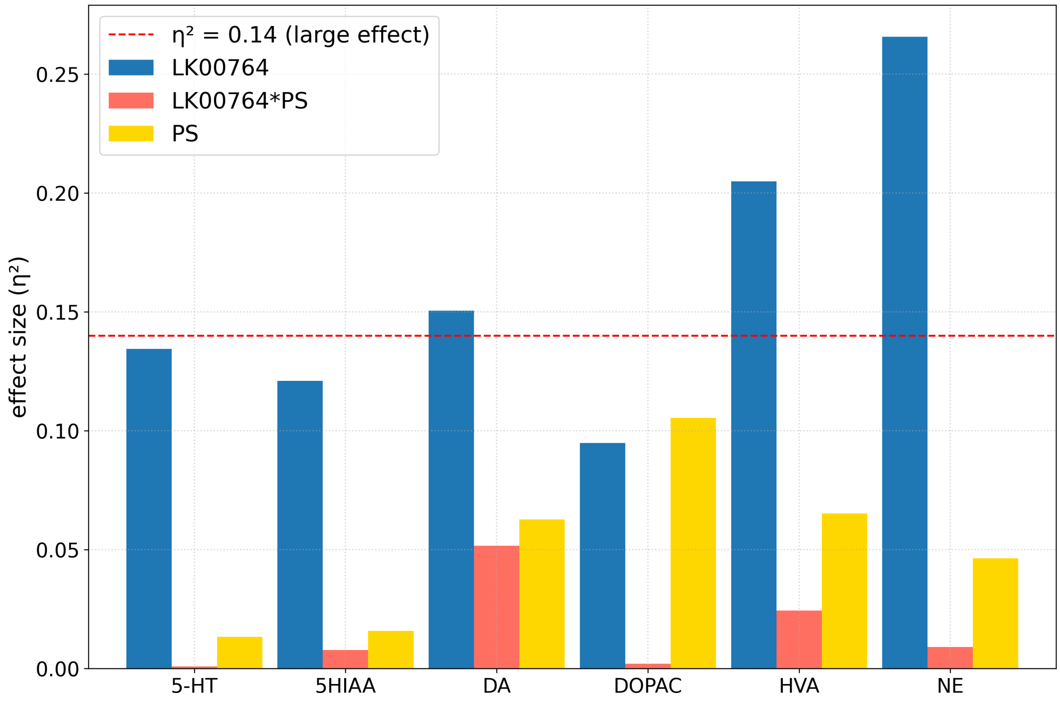

Figure S7. Effect size (η^2) computed from two-way ANOVA for neurotransmitter and metabolite concentrations in the striatum. Bars illustrate the magnitude of main effects (treatment and stress) and their interaction. The dashed red line denotes a large effect threshold ($\eta^2 = 0.14$).

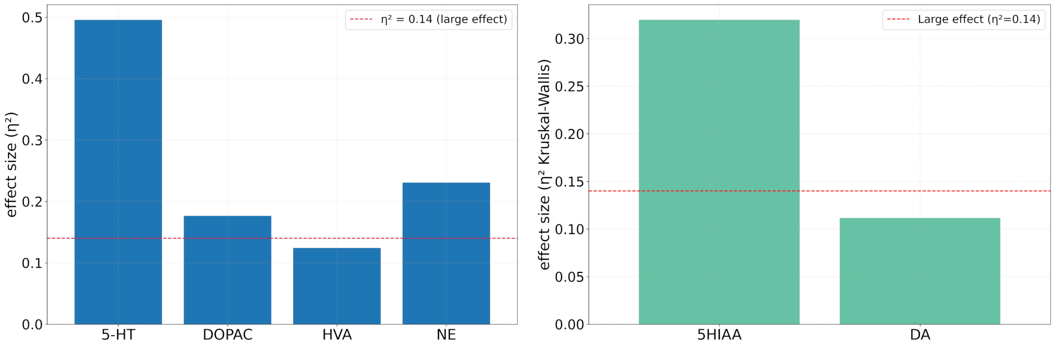

Figure S8. Neurotransmitter and metabolite levels in the hippocampus across experimental groups. Left panel shows results of ANOVA with effect size (η^2), and right panel shows results of the Kruskal–Wallis test. The dashed red line denoting a large effect threshold ($\eta^2 = 0.14$).

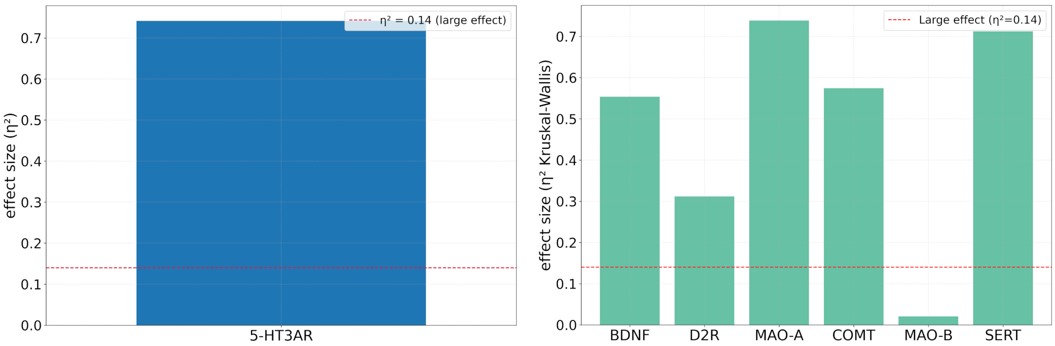

Figure S9. Large effect sizes in striatal expression of monoaminergic and neurotrophic genes in PTSD rats (η^2 and η^2_{KW}). Effect size values were derived from the ANOVA and Kruskal–Wallis tests.

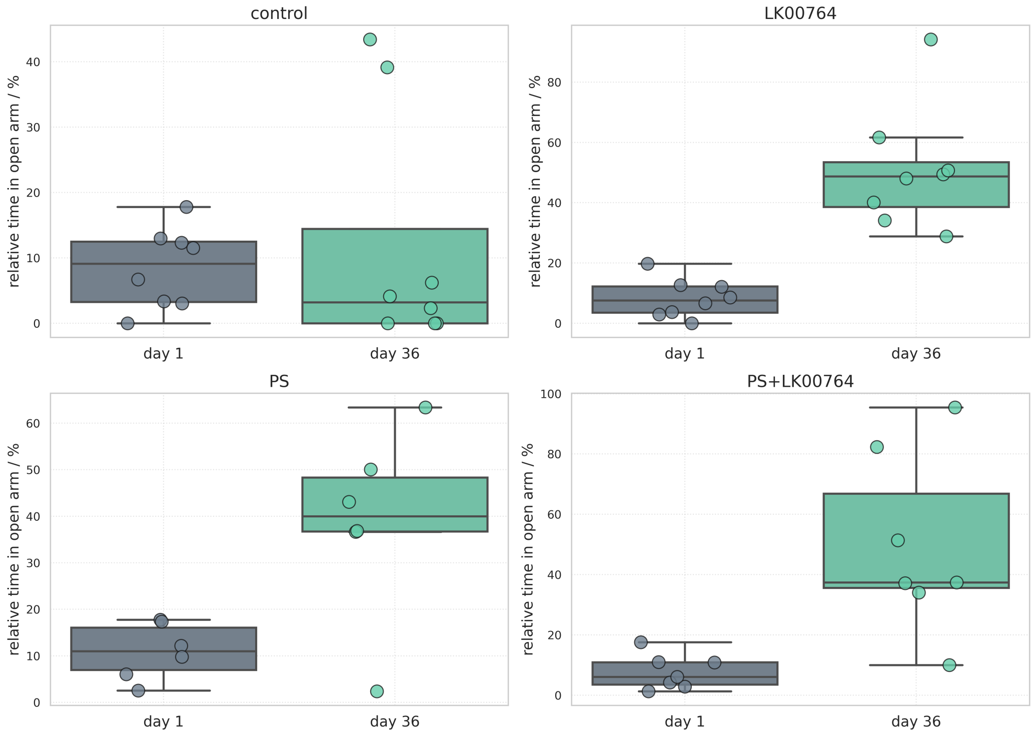

Figure S10. Relative open-arm exploration in the elevated plus maze at baseline (day 1) and following chronic stress exposure and/or treatment (day 36). Data are shown for the control, LK00764-treated, predator stress (PS), and combined PS+LK00764 groups, expressed as percentage of total arm exploration time.

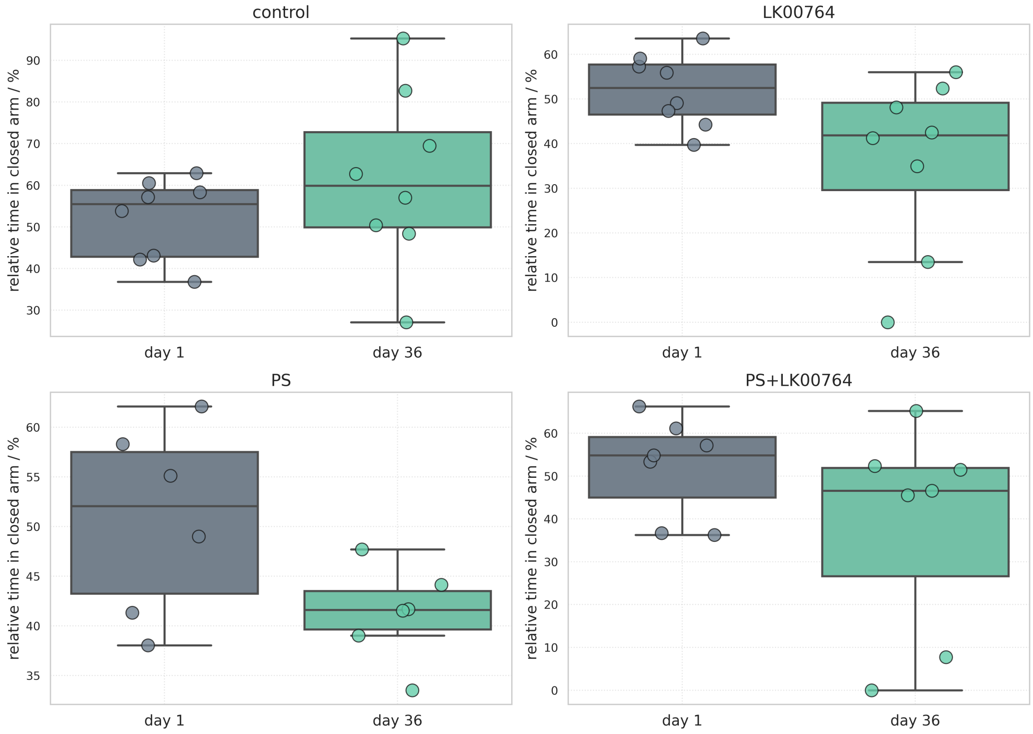

Figure S11. Relative time spent in the closed arms of the elevated plus maze at baseline (day 1) and after chronic stress exposure and/or treatment (day 36). Values are presented for the control, LK00764-treated, predator stress (PS), and combined PS+LK00764 groups, expressed as a percentage of total arm exploration time.

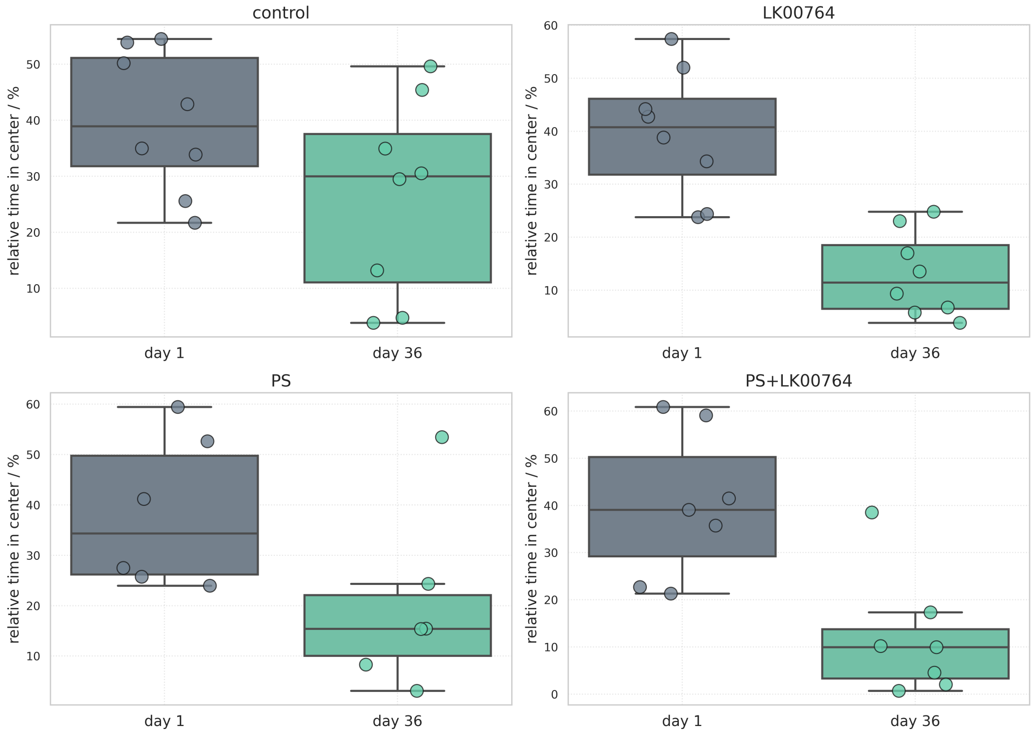

Figure S12. Relative exploration of the central area in the elevated plus maze at baseline (day 1) and following chronic stress exposure and/or treatment (day 36). Data are provided for the control, LK00764-treated, predator stress (PS), and combined PS+LK00764 groups, expressed as a percentage of the total time spent in all maze zones.
